# Recent Advances in Electrode Design for Rechargeable Zinc–Air Batteries

**DOI:** 10.1002/smsc.202100044

**Published:** 2021-08-06

**Authors:** Jinfa Chang, Guanzhi Wang, Yang Yang

**Affiliations:** ^1^ NanoScience Technology Center University of Central Florida 12424 Research Parkway Suite 423 Orlando FL 32826 USA; ^2^ Department of Materials Science and Engineering University of Central Florida Orlando FL 32826 USA; ^3^ Department of Chemistry Renewable Energy and Chemical Transformation Cluster University of Central Florida Orlando FL 32826 USA

**Keywords:** air cathodes, bifunctional catalysts, electrode design, metal anodes, zinc–air batteries

## Abstract

Rechargeable zinc–air batteries (ZABs) show enticing prospects as next‐generation energy conversion and storage technology due to their unique merits of environmental friendliness, low cost, impressive energy density, and high security. However, the dendrite growth, surface passivation, and metal anode corrosion, as well as the sluggish reaction kinetics, deficient bifunctionality, high platinum group metals (PGMs) dependence, and corrosion of carbon‐based materials for air cathodes, are the main problems hindering the large‐scale application of ZABs. Herein, the fundamental principles of ZABs are first introduced. The detailed discussions will be focused on the electrochemical aspects of the metal anode and air cathode by making a comprehensive comparison of the recent progress in the field. Lastly, brief perspectives on the further development of rechargeable ZABs are introduced. This review aims to provide a better understanding of electrode design for ZABs, which will provide guidelines for the design and fabrication of high‐performance and cost‐effective ZABs.

## Graphical abstract

This review summarizes the latest progress in electrochemistry and material science of zinc–air batteries (ZABs). The fundamentals and current challenges of Zn anodes and air cathodes are outlined and the corresponding insightful solutions such as novel electrode design and interface engineering are proposed. Finally, a brief perspective on the further development of high‐performance and cost‐effective rechargeable ZABs is overviewed.

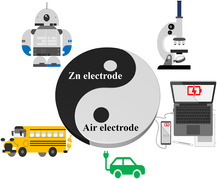

## 1 Introduction

With the speedy development of contemporary society and industry, severe energy and environmental crises are looming.^[^ ^1^ ^]^ To cope with the ongoing issues, more attention has been distracted from traditional fossil fuels to sustainable and green chemical energy sources such as fuel cells and batteries,^[^ ^2^ ^]^ which are low‐carbon emission and eco‐friendly with high energy density.^[^ ^3^ ^]^ Particularly, lithium‐ion batteries (LIBs) are leading to the rechargeable battery markets with wide applications in our everyday life, such as smart phones, laptops, electric vehicles, smart grids, and mobile power supplies.^[^ ^4^ ^]^ However, the high cost, safety risk from the Li combustibility, limited energy density, and the potential supply shortage of Li severely impede the further development of LIBs. Therefore, it is urgent to explore and develop more efficient and safer energy storage devices.^[^ ^5^ ^]^

**Figure 1:**
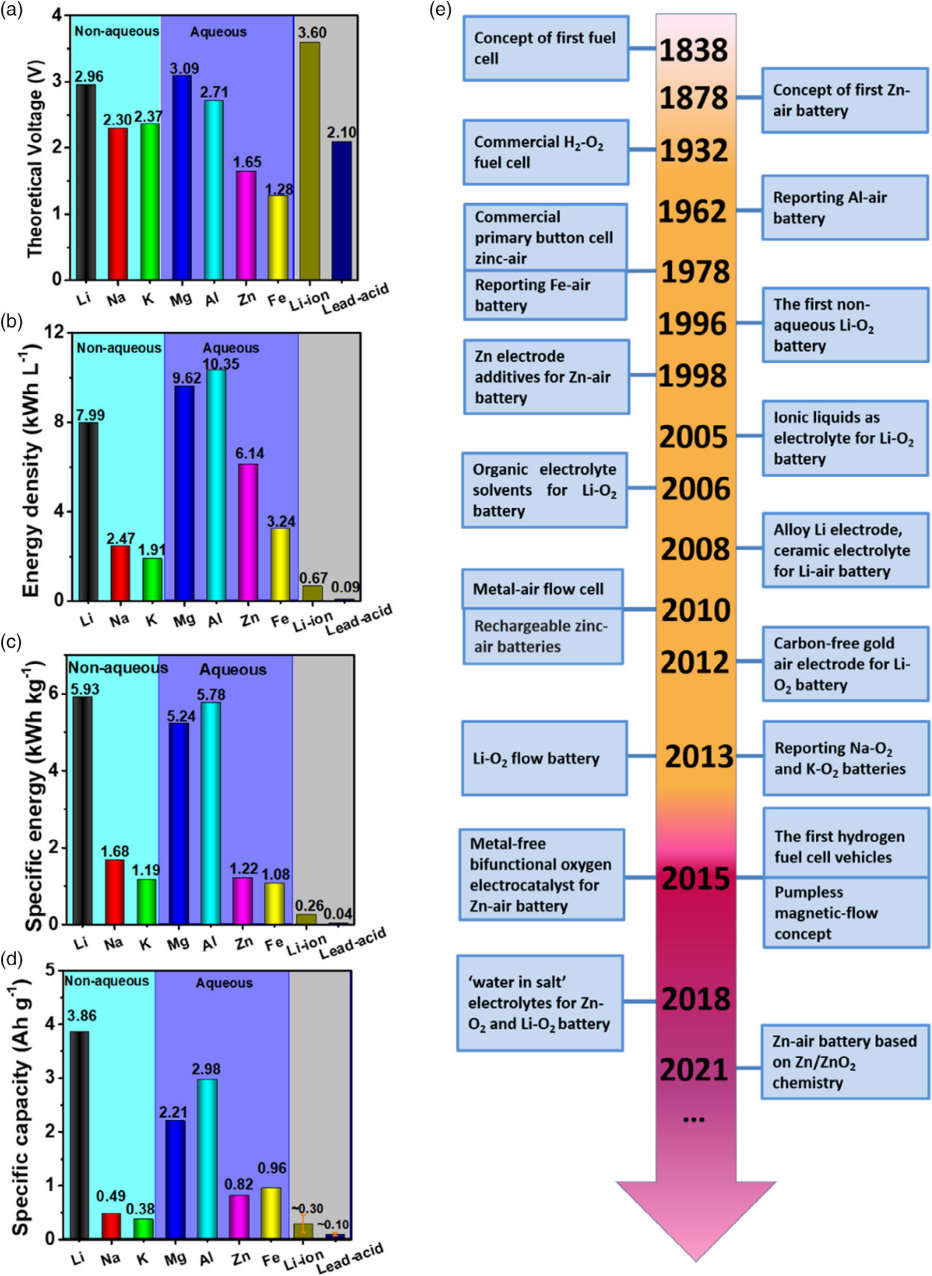
Comparisons of a) theoretical voltage, b) energy density, c) specific energy, and d) specific capacity between metal–air batteries, lead–acid batteries, and lithium‐ion batteries. The detailed values were calculated from the literature.^6^, ^7^, ^97^ e) Roadmap for metal–air batteries and fuel cells.

Metal–air batteries (MABs) contain anode metals with high valence electron to atomic nuclei ratio (such as Li, Na, K, Mg, Al, Zn, and Fe), and unique open cell structure with the cathode oxygen (O_2_) from external infinitely ambient, which have attracted great attention as emerging electrochemical energy storage devices due to their cost‐effectiveness and high energy density.^4^, ^6^ The theoretical voltage, specific energy density, volumetric energy density, and specific capacity of various MABs are compared in **Figure** **1**a–d.^6^, ^7^ Due to the lightweight nature of Li, the Li–air (oxygen) batteries can theoretically furnish the highest specific energy density, as well as high battery voltage. However, the MABs based on alkali metals (i.e., Li, Na, and K) are very sensitive to water and moisture, making them unsafe in aqueous environments. However, the low coulombic efficiency and self‐discharge of these nonaqueous MABs limit their practical applications. Mg–air and Al–air batteries can also offer comparable theoretical energy densities and working voltages as Li–air batteries. Nevertheless, the serious parasitic corrosion resulted from the hydrogen evolution reaction (HER) on Mg and Al electrodes surfaces and the nonrechargeable feature caused by thermodynamical infeasibility of the Al and Mg electrodeposition in aqueous electrolyte restrict their widespread applications. On the contrary, both Fe–air and Zn–air batteries (ZABs) can be recharged under aqueous environments. ZABs possess peerless advantages such as higher energy density and theoretical voltage under alkaline conditions than Fe–air batteries (Figure 1a–d).^[^ ^8^ ^]^ Currently, the primary ZABs have been commercially used as hearing‐aid batteries (Figure 1e),^[^ ^9^ ^]^ and some companies such as EOS Energy Storage, FluidicEnergy, and ZincNyx have been committed to developing ZABs as grid energy storage systems.^6^, ^7^, ^8^, ^10^ Given the distinct advantages of ZABs over others, this review will be focused on the ZABs.

To improve the practical energy density of ZABs for extensive application, there are still some scientific and technical problems to be solved. The critical issues at the Zn anode are mainly related to the dendrite growth and the resulted membrane piercing, self‐discharge, and fast metal anode consumption.^[^ ^11^ ^]^ For the air cathodes, the deficient bifunctional activities and sluggish kinetics for oxygen reduction reaction (ORR) and oxygen evolution reaction (OER), as well as the corrosion problems of carbon‐based materials, are the major concerns.^6^, ^7^, ^10^, ^12^ Herein, we conduct a comprehensive review of ZABs from fundamental electrochemical aspects and also summarize the current issues in the development of ZABs. First, we will briefly introduce the fundamentals of ZABs, including the origin of the proposed concept, the structural composition, and the working principle. Then the application challenges of Zn anode and air cathode in ZABs are emphatically reviewed. The achievements that have been made and the existing limitations of ZABs will be summarized and discussed. Lastly, brief perspectives on the development of ZABs are suggested.

## 2 Working Principles

**Figure 2:**
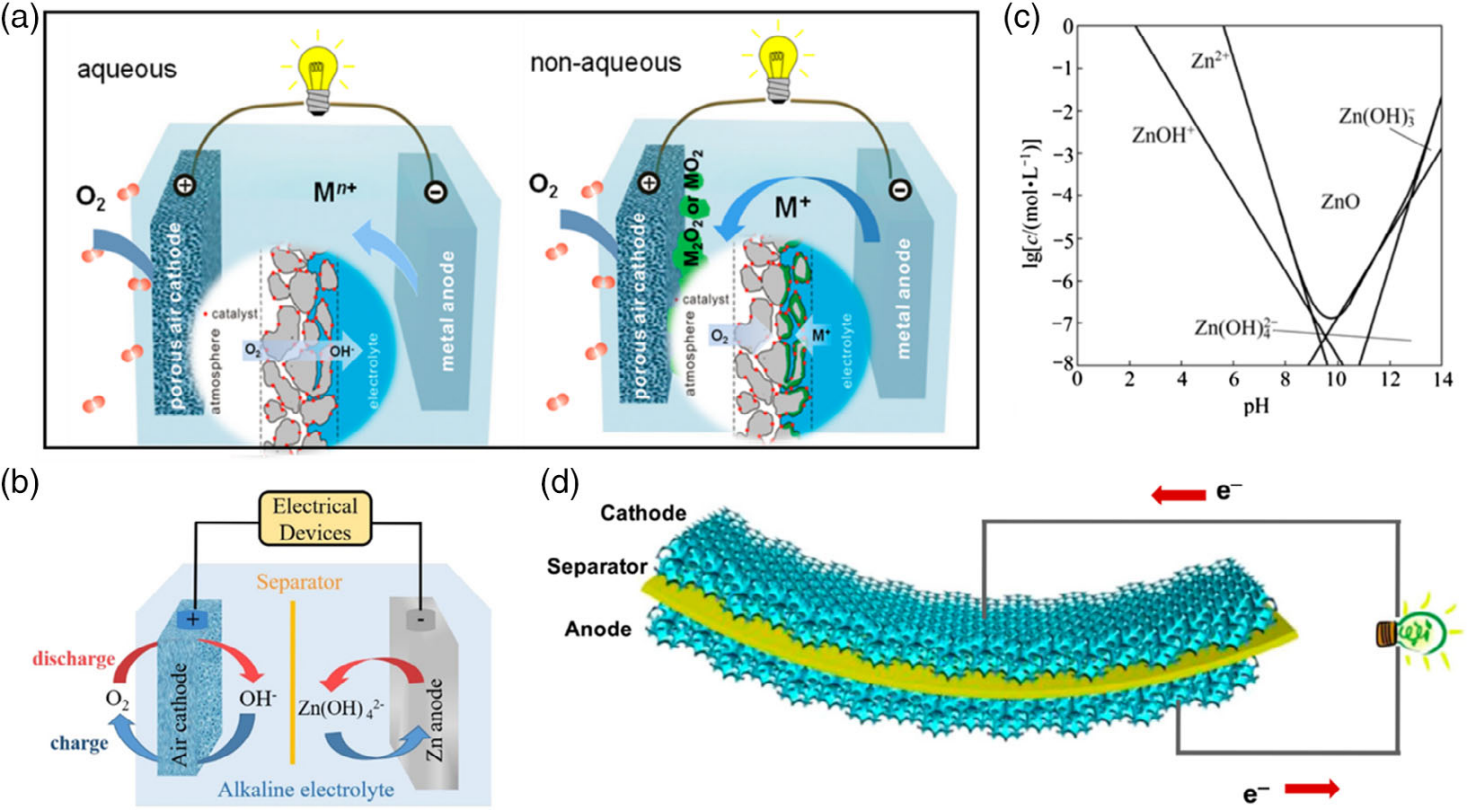
a) Typical cell structures of aqueous and nonaqueous stationary metal–air batteries. Reproduced with permission.^[^ ^97^ ^]^ Copyright 2017, American Chemical Society. b) A typical rechargeable ZABs and discharge/charge process. Reproduced with permission.^[^ ^17^ ^]^ Copyright 2018, Royal Society of Chemistry. c) Solubility of Zn species as a function of pH at 25 °C. Reproduced with permission.^[^ ^98^ ^]^ Copyright 2010, Springer Nature. d) Schematic of a flexible ZAB. Reproduced with permission.^[13b]^ Copyright 2018, National Academy of Sciences.

As a typical family member of MABs powered by metal oxidation and oxygen reduction during discharging, the concept of ZABs was first proposed in 1878,^[^ ^9^ ^]^ and then extended rapidly to other metal anodes‐based MABs, such as Al, Fe, Li, Mg, Na, K, and so on (Figure 1e). The operating environment of MABs is broadly categorized into aqueous (alkaline KOH and NaOH solution) and nonaqueous systems for nonalkali metals and alkali metals anodes, respectively (**Figure** **2**a). These MABs have different theoretical open‐circuit voltages, specific energy densities, specific volumetric energy densities, and specific capacities (Figure 1a–d), which should be considered when using for different purposes.

In principle, primary stationary ZABs are composed of metal anodes, separators soaked with electrolytes, and air cathodes (Figure 2b). The separator is mainly used to separate anodes/cathodes and prevent the dendrite‐induced short circuit of ZABs. During the discharge process, the Zn anode will lose two electrons and be oxidized to Zn^2+^, which then react with OH^−^ in the alkaline electrolyte to form Zn(OH)_4_^2−^ (Equation (1) and Figure 2b). Zn(OH)_4_^2−^ will be decomposed into insoluble and semiconducting Zn oxide (ZnO) when the concentration reaches supersaturation in the electrolyte (Equation (2) and Figure 2c). In the meantime, two liberated electrons from the anode transfer to the air cathode through the external circuit and reduce O_2_ to hydroxide ions (OH^−^) at the cathode (ORR, Equation (3)). Overall, a theoretical potential of 1.65 V (Figure 1a) can be delivered. When recharging ZABs (reverse reactions of Equation ((1), (2), (3))), the deposition of Zn from Zn^2+^ dissolved in the electrolyte occurs on the metal anode and OER occurs at the air cathode.

(1) $$\text{Zn}+4{\text{OH}}^{-}\overset{\text{discharge}}{\to }\text{Zn}{\left(\text{OH}\right)}_{4}^{2-}+2e^{-}$$

(2) $$\text{Zn}{\left(\text{OH}\right)}_{4}^{2-}\to \text{ZnO}+H_{2}O+2{\text{OH}}^{-}$$

(3) $$O_{2}+4e^{-}+2H_{2}O\overset{\text{discharge}}{\to }4{\text{OH}}^{-}$$

Taking advantage of low cost, inherent security, high theoretical energy storage density, flat discharge voltage, nonreactivity, and flexibility of zinc metal compared with other systems, ZABs are excellent candidates for flexible power sources (Figure 2d).^6^, ^13^ Due to the special requests for flexibility, the liquid electrolyte cannot be used in flexible ZABs because the electrolyte leakage and evaporation could damage sensitive electronic equipment. Thus, the major research on the flexible ZABs is focused on the development of solid‐state electrolyte which possesses sufficient ionic conductivity and mechanically robust electrodes when working under deformations (**Table** **1**). However, the performance of flexible ZABs using solid‐state electrolytes is always inferior to the ZABs using liquid electrolytes due to their lower ionic conductivity. Much attention should be paid to the development of solid‐state electrolytes with low resistance and high (electro)chemical stability during the operation. 6, 13, 14

**Table 1:** Summary of recently typical Zn electrode and air electrode performance for static ZABs, zinc–air flow cells, and all‐solid‐state ZABs

Anode	Electrolyte	Air electrode	ORR half‐wave potential/OER performance (V vs RHE)	OCV [V]	Power density [mW cm^−2^]	Specific energy density [mAh g_Zn_^−1^)	Stability @ current density	Ref.
Zn foil	6 m KOH + 0.2 m Zn(ac)_2_	FeNi‐SAs@NC	*E*_ORR1/2_ = 0.907 V	1.54	260; 70 (all‐solid‐sate)	950	20 mA cm^−2^, 100 h	[102]
			*E*_OERj=10_ = 1.528 V					
Zn foil	6 m KOH and 0.2 m ZnCl_2_	Pt/Fe‐NC	*E*_ORR1/2_ = 0.839 V	1.42	178.9	763.6	20 mA cm^−2^, 76.4 h	[58c]
			Unknown for OER					
Zn foil	6 m KOH + 0.2 m ZnCl_2_	IOSHs‐NSC‐Co_9_S_8_	*E*_ORR1/2_ = 0.83 V	1.497	133	768	240 cycles (80 h)	[14d]
			*E*_OERj=10_ = 1.64 V					
Zn foil	6 m KOH + 0.2 m Zn(ac)_2_	PBA‐Pd‐Co/C	*E*_ORR onset_ = 0.90 V	–	138.8	471.6	5 mA cm^−2^, 167 h (500 cycles)	[58i]
			*E*_OERj=10_ = 1.85 V					
Zn foil	6 m KOH + 0.2 m Zn(ac)_2_	SA‐PtCoF	*E*_ORR onset_ = 0.95 V	1.31	125	808	10 mA cm^−2^, 260 h	[55g]
			*E*_OER onset_ = 1.50 V					
Zn foil	6 m KOH	PtCo@NMC	*E*_ORR1/2_ = 0.94 V	1.426	189	–	5 mA cm^−2^, 26 h	[58b]
			Unknown for OER					
Zn foil	6 m KOH + 0.2 m Zn(ac)_2_	O‐Pd‐Fe@NC/C	*E*_ORR1/2_ = 0.91 V	1.43	169	779	5 mA cm^−2^, 160 h	[58g]
			Unknown for OER					
Zn foil	6 m KOH	FexN	*E*_ORR1/2_ = 0.885 V	–	180	668	5 mA cm^−2^, 60 h	[103]
			Unknown for OER					
Zn foil	6 m KOH + 0.2 m Zn(ac)_2_	CoP/NC‐800	*E*_ORR1/2_ = 0.78 V	1.404	114	695.08	10 mA cm^−2^, 35 h	[104]
			*E*_OERj=10_ = 1.52 V					
Zn foil	6 m KOH + 0.2 m Zn(ac)_2_	(Co,Fe)_3_N_2D	Δ*E*_j=30_ = 0.85 V	–	234	–	30 mA cm^−2^, 300 h	[105]
Zn foil	6 m KOH + 0.2 m ZnCl_2_	Co/CNFs (1000)	*E*_ORR1/2_ = 0.896 V	–	–	–	15 mA cm^−2^, 200 h	[66a]
			*E*_OERj=10_ = 1.55 V					
Zn foil	O_2_‐saturated 6 m KOH	Fe SAs/N‐C	*E*_ORR1/2_ = 0.91 V	–	225	636	10 mA cm^−2^, 260 h	[106]
			Unknown for OER					
Zn foil	6 m KOH + 0.2 m Zn(ac)_2_	FeNiP/NCH	*E*_ORR1/2_ = 0.75 V	1.48	250	–	10 mA cm^−2^, 500 h (2100 cycles)	[107]
			*E*_OERj=10_ = 1.48 V					
Zn foil	6 m KOH + 0.2 m Zn(ac)_2_	Pt‐SCFP/C‐12	*E*_ORR1/2_ = 0.81 V	1.40	122	790.4	5 mA cm^−2^, 80 h (240 cycles)	[58a]
			*E*_OERj=10_ = 1.60 V					
Zn foil	6 m KOH	Pd/F, N‐doping G	*E*_ORR1/2_ = 0.87 V	1.40	229	–	10 mA cm^−2^, 36 h	[58f]
			Unknown for OER					
Zn foil	6 m KOH + 0.2 m Zn(ac)_2_	PdMo bimetallene/C	*E*_ORR1/2_ = 0.95 V	1.483	154.2	798	10/50/75 mA cm^−2^, 350 h	[55f]
			*E*_OERj=10_ = 1.71 V					
Zn foil	–	Fe—N—S CNN	*E*_ORR1/2_ = 0.91 V	1.37	132	700	20 mA cm^−2^, 13 h	[108]
			Unknown for OER					
Zn foil	6 m KOH + 0.2 m Zn(ac)_2_	CoFe20@CC	*E*_ORR1/2_ = 0.86 V	1.50	190.3	789.7	5 mA cm^−2^, 400 cycles	[109]
			*E*_OERj=10_ = 1.657 V					
Zn foil	6 m KOH + 0.2 m Zn(ac)_2_	Co SAs@NC	*E*_ORR1/2_ = 0.82 V	1.46	105.3	897.1	1 mA cm^−2^, 1000 min (flexible solid‐state)	[70f]
			Unknown for OER	1.40 (flexible)				
Zn foil	6 m KOH + 0.2 m Zn(ac)_2_	NCo@CNT‐NF700	*E*_ORR1/2_ = 0.861 V	1.460	220	–	5 mA cm^−2^, 130 h (400 cycles)	[66c]
			Unknown for OER					
Zn foil	6 m KOH	Fe—N/C‐700	*E*_ORR1/2_ = 0.863 V	1.53	73	707	25 mA cm^−2^, 28 h	[110]
			Unknown for OER					
Zn foil	6 m KOH + 0.2 m Zn(ac)_2_	C, O‐PdFe@Pt/C	*E*_ORR1/2_ = 0.85 V	–	293	–	5 mA cm^−2^, 124 h	[58e]
			Unknown for OER					
Zn foil	6 m KOH + 0.2 m Zn(ac)_2_	NiCo/NLG‐270	*E*_ORR1/2_ = 0.82 V	1.49	103	403	20 mA cm^−2^, 15 h (40 cycles)	[111]
			*E*_OERj=10_ = 1.57 V					
Zn sheets	6 m KOH	Ti_0.8_Co_0.2_N	*E*_ORR onset_ = 0.96 V	1.42	130.9	1590	5 mA cm^−2^, 46 h	[112]
			Unknown for OER					
Zn foil	6 m KOH + 0.2 m ZnCl_2_	Fe‐N_4_SAs/NPC	*E*_ORR1/2_ = 0.885 V	–	232	–	2 mA cm^−2^, 36 h (108 cycles)	[70d]
			*E*_OERj=10_ = 1.66 V					
Zn foil	6 m KOH	CoP	*E*_ORR1/2_ = 0.858 V	1.34	61	–	30 mA cm^−2^, 12 h	[113]
			*E*_OERj=10_ = 1.51 V					
Zn foil	6 m KOH + 0.2 m ZnCl_2_	N,P/CoS_2_@TiO_2_NPFs	*E*_ORR onset_ = 0.91 V	1.317	–	610	130 h	[79]
			*E*_OER onset_ = 1.41 V					
Zn foil	6 m KOH + 0.2 m Zn(ac)_2_	Pd/B_4_C	*E*_ORR onset_ = 0.96 V	–	187	–	5 mA cm^−2^, 1333 h (4000 cycles)	[58d]
			*E*_OERj=10_ = 1.75 V					
Zn foil	6 m KOH + 0.2 m Zn(ac)_2_	Pd/FeCo	*E*_ORR1/2_ = 0.85 V	1.42	117	821	10 mA cm^−2^, 200 h (400 cycles)	[58n]
			*E*_OERj=10_ = 1.55 V					
Zn foil	6 m KOH + 0.2 m ZnCl_2_	Pd‐coated CoFe composite/NCNTs	*E*_ORR onset_ = 0.897 V	1.479	261	–	10 mA cm^−2^ for 50 h	[58j]
			*E*_OERj=10_ = 1.515 V					
Zn foil	8 m KOH + 0.5 m ZnO	NiS_*x*_‐FeO_*y*_/SCFP	*E*_ORR1/2_ = 0.78 V	–	–	–	20 mA cm^−2^, 110 h (55 cycles)	[114]
			*E*_OERj=10_ = 1.60 V					
Zn foil	PAM‐co‐PAA gel/6 m KOH	IOSHs‐NSC‐Co_9_S_8_	*E*_ORR1/2_ = 0.83 V	1.408	60	738	105 cycles/35 h	[14d]
			*E*_OERj=10_ = 1.64 V					
Zn foil	6 m KOH + 0.2 m Zn(ac)_2_	Pd/Co(OH)_2_	*E*_ORR1/2_ = 0.87 V	1.40	–	766	5 mA cm^−2^, 185 h	[58h]
			*E*_OERj=10_ = 1.60 V					
Za foil	PVA/KOH/Zn(ac)_2_	1 nm COOx	*E*_ORR1/2_ = 0.896 V	1.39	–	300 W gcat^−1^	6 mA cm^−2^, 10 h	[13d]
			*E*_OERj=10_ = 1.60 V					
Zn foil	(PVA) gel electrolyte	Co_3_O_4_@N‐CNMAs/CC	*E*_ORR1/2_ = 0.90 V	1.461	65	815	5 mA cm^−2^, 385 h	[115]
			*E*_OERj=10_ = 1.54 V					
Zn foil	PVA/KOH/ZnAc_2_	Ni,N codoped np‐graphene	*E*_ORR1/2_ = 0.85 V	1.35	83.8	–	2 mA cm^−2^; 258 cycles (43 h)	[116]
			*E*_OERj=10_ = 1.50 V					
Zn foil	(ac)_2_	CoNi@NCNT/NF	*E*_ORR1/2_ = 0.87 V	1.40	127	655	5 mA cm^−2^, 90 h	[65]
			*E*_OERj=10_ = 1.54 V					
Zn foil	6 m KOH + 0.2 m Zn(ac)_2_	CoS*x*@PCN/rGO	*E*_ORR1/2_ = 0.78 V	1.38	–	–	50 mA, 43.8 h	[61]
			*E*_OERj=10_ = 1.57 V					
Zn plate	–	NiS_*x*_ FHF	*E*_ORR onset_ = 0.86 V	–	–	–	2 mA cm^−2^, 3000 min	[13h]
			*E*_OERj=10_ = 1.56 V					
Zn foil	6 m KOH + 0.2 m Zn(ac)_2_M for aqueous; PVA/KOH for all solid state	NiO/CoO TINWs	*E*_ORR1/2_ = 0.818 V	1.492 V for aqueous. 1.354 V for all‐solid‐state	151	842.58@25 °C	2 mA cm^−2^, 100 cycles (332 h)	[14a]
			*E*_OERj=10_ = 1.50 V			328.57@‐10 °C		
						313.28@80 °C		
Zn foil	Alkalified‐PAA	1D BFCs	*E*_ORR1/2_ = 0.90 V	1.49@22^o^C	160	721@0 °C; 691@−20 °C	5 mA cm^−2^, 400 cycles	[13g]
			*E*_OERj=10_ = 1.60 V	1.44@0 °C	122.9			
				1.40@‐20 °C	80.5			
Zn_3_Mn	2 m ZnSO_4_ in seawater	Pt/C‐RuO_2_	Unknown	–	196	816.3	80 mA cm^−2^, 1900 cycles.	[11b]
Zn foil	(Zn(OTf)_2_	Carbon black	Unknown	–	–	–	0.4 mA cm^−2^, 300 h; 0.1 mA cm^−2^, 1600 h; 1.0 mA cm^−2^, 160 h	[90]
Zn particle/Ni mesh	40% wt. KOH	Mn/C	Unknown	–	–	–	55–65 °C, flow velocity of 0.12 m s^−1^	[117]
Zinc grain	O_2_‐saturated KOH	Ag/C	Unknown	–	–	34/72	–	[22f]
Zinc springs	PANa‐cellulose hydrogel	Fe/N/C	Unknown	1.47	210.5	–	5 mA cm^−2^ 55 h (300 cycles)	[118]
3D Zn sponge + 300 ppm (In + Bi)	6 m KOH/PPA	Carbon/cryptomelane/Teflon	Unknown	Unknown	Unknown	728	89% ZUR; 40% depth of discharge; 5 mA cm^−2^ 90 h	[11a]
3D Zn/Cu foam	8 m KOH + 0.5 m ZnO	Commercial Ni(OH)_2_	Unknown	Unknown	286	754	92% ZUR; 200 mA cm^−2^, 10 000 cycles	[119]
3D hyperdendritic zinc sponge/Ni foam	8.9 m KOH + 0.61 m ZnO	Unknown	Unknown	Unknown	Unknown	719	88% ZUR; C/5 rate, 100 cycles	[23]
Calcium zincates powder + 5 wt% zinc + 10 wt% acetylene black + 5 wt% PTFE	6 m KOH + Saurated ZnO	Commercial Ni(OH)_2_	Unknown	Unknown	Unknown	385	98% ZUR; 2C rate, 250 cycles	[120]
Bi‐coated ZnO powder + 4.8 wt% PTFE + 0.6 wt% CMC	4.5 m KOH + 1.6 m K_2_BO_3_ + 0.9 m KF + 0.1 m LiOH	Commercial Ni(OH)_2_	Unknown	Unknown	Unknown	656	90% ZUR; C/5 rate, 50 cycles	[121]
In‐doped ZnO powder + 8.3 wt% PTFE + 8.3 wt% graphite	4.5 m KOH + 1.0 m NaOH + 0.5 m LiOH + Saturated ZnO	Commercial Ni(OH)_2_	Unknown	Unknown	Unknown	586	92% ZUR; C/4 rate,73 cycles	[28b]
Zn–Al‐layered double oxide powder + 10 wt% acetylene black + 5 wt% PTFE	6 m KOH + Saturated ZnO	Commercial Ni(OH)_2_	Unknown	Unknown	Unknown	469	87% ZUR; 1 C rate, 1000 cycles	[122]
PANI‐coated Zn	ADE‐75	7 m KOH solution + 2 wt% polyacrylic acid	Unknown	Unknown	Unknown	565.3	96.9% Capacity retention	[123]
0.5 wt% CuO—Zn gels	Unknown	10 m KOH	Unknown	Unknown	Unknown	Unknown	65 mA g^−1^, 19 h	[124]
Zn_3_(PO_4_)_2_ + ZnF_2_ + KPF_6_	MnO2/CNT + 20 wt% Super P + 10 wt% PVP	2 m ZnSO_4_ + 0.05 m KPF_6_	Unknown	Unknown	Unknown	Unknown	5–10 mA cm^−2^, 808 h	[36a]
Zn@Nafion—Zn—X	Zn plate	2 m ZnSO_4_	Unknown	Unknown	Unknown	Unknown	Coulombic efficiency 97%; 10 mAh cm^−2^, 10 000 cycles	[35]
MOF—PVDF‐coated Zn	MnO_2_ + 20 wt% acetylene black + 10 wt% PVDF	3 m ZnSO_4_ + 0.1 m MnSO_4_	Unknown	Unknown	Unknown	Unknown	3 mA cm^−2^, 500 cycles	[37]
Polymer modified Zn	α‐MnO_2_ + 20 wt% Super P + 10 wt% PVDF	2 m ZnSO_4_ + 0.1 m MnSO_4_	Unknown	Unknown	Unknown	Unknown	95.1% Coulombic efficiency; 0.5 mA cm^−2^, 8000 h	[36e]
SEI‐coated Zn	V_2_O_5_ + 20 wt% Super P + 10 wt% PVDF	Acetamide‐Zn(TFSI)_2_	Unknown	Unknown	Unknown	Unknown	≈100% Coulombic efficiency; 0.1 mA cm^−2^, 1000 h	[36b]
Zn power +10.% carbon black +10% PVDF	Porous carbon	1 m Zn(TFSI)_2_ + 20 m LiTFSI	Unknown	Unknown	Unknown	Unknown	3000 mAh g^−1^ at 0.9 V discharge potential; 200 cycles	[29]
Carbon‐coated Zn	NVPF@C	8 m NaClO_4_ + 0.4 m Zn(CF_3_SO_3_)_2_	Unknown	Unknown	Unknown	Unknown	≈100% Coulombic efficiency; 2 A g^−1^, 1000 cycles	[32d]
Nanoporous CaCO_3_ coating Zn foil	CNT/MnO_2_	3 m ZnSO_4_ + 0.1 m MnSO_4_	Unknown	Unknown	Unknown	177 mAg g^−1^ after 1000 cycles; 124 mAh g^−1^ without coating	0.25 mA cm^−2^, 800 h	[34]

## 3 Chemistry and Materials of Anodes

Due to the unlimited O_2_ source from the atmosphere to the air cathodes, the capacity and performance of ZABs are mostly dependent on the utilization efficiency of anodes.^[^ ^15^ ^]^ Commercial Zn in various forms (i.e., plates/foils/powders, etc.) have been widely used in fundamental studies of ZABs due to their low toxicity, low weight, earth‐abundance, high capacity, and better stability in an aqueous alkaline electrolyte than other metals used for MABs such as Al. The shape, morphology, and surface area of Zn have significant influences on the ZABs performance, which will affect the interparticle contact, internal electrical resistance, and electrochemical performance. Different morphologies, such as spheres, ribbons, flakes, dendrites, foams, and fibers of Zn anodes, have been widely explored and used for ZABs.^[^ ^16^ ^]^ However, the corrosion rate of Zn anodes increases with the surface area because of the accelerated side reactions at the interfaces, consuming the electrolyte, reducing the utilization efficiency of Zn, and finally shortening the lifetime of ZABs. Generally, the side reactions occurring on the Zn anode during the cycling of ZABs may include dendrite growth, surface passivation, shape change, and HER on the Zn electrode, which reduce the Coulombic efficiency (CE) of ZABs.

### 3.1 Dendrite Growth and Surface Passivation on the Zn Anode

**Figure 3:**
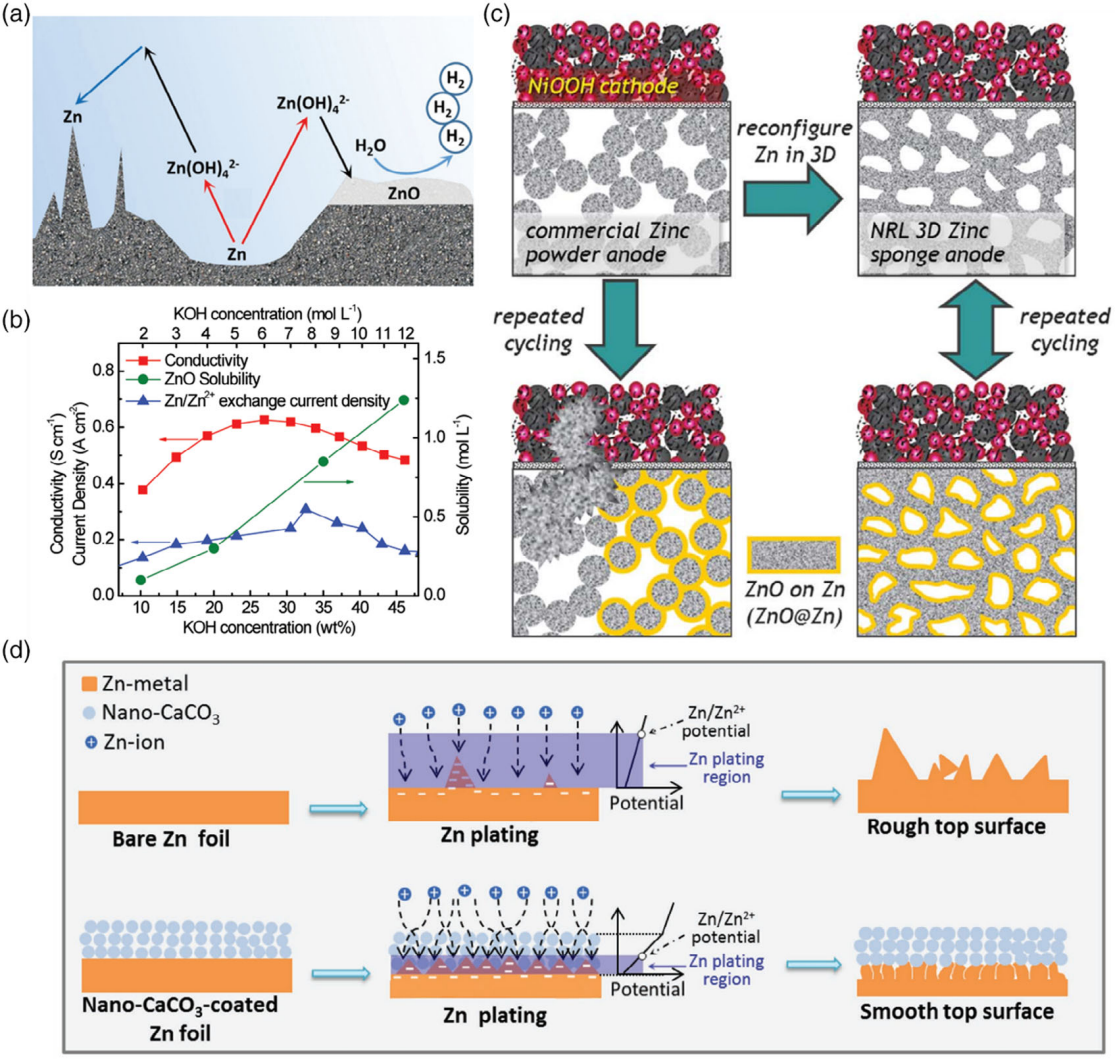
a) Schematic of dendrite growth, surface passivation, and hydrogen evolution (from left to right), on the surface of the Zn anode. b) Conductivity, solubility, and Zn/Zn^2+^ exchange current density as a function of KOH concentration. Reproduced with permission.^[6a]^ Copyright 2017, Wiley. c) The impacts of 3D sponge structure on the reversibility of the Zn anode. Reproduced with permission.^[^ ^31^ ^]^ Copyright 2017, American Association for the Advancement of Science. d) Illustrations of morphology evolution for bare and CaCO_3_‐coated Zn foils during Zn stripping/plating cycling. Reproduced with permission.^[^ ^34^ ^]^ Copyright 2018, Wiley.

The nucleation and growth of Zn dendrites occur on the surface of the Zn anode during ZABs charging (plating).^[^ ^17^ ^]^ The nuclei of Zn do not necessarily form the site where they were consumed during discharging (stripping, **Figure** **3**a), which results in the formation of metallic Zn protrusions with destructive effects on the cyclability of ZABs. The dendrite growth will not only lead to the performance decay of ZABs but also cause an internal short circuit due to the membrane pierce by the dendritic crystal. Recent studies suggest that the inhomogeneous distribution of local current and electro‐osmotic forces across the ZABs caused by the convective ﬂows can lead to the shape reconstruction of the Zn anode.^[6a]^ On the contrary, the shape reconstruction of Zn will further change the intensity of the local electric field result in the progressive acceleration of dendrite growth and capacity loss. It is found that both the Zn anode and air cathode show superior activity because of the lower viscosity, maximum ion conductivity, and higher oxygen diffusion coefficients in the electrolyte of 6–7 m (25–30%) KOH solution (Figure 3b). However, the Zn/Zn^2+^ exchange current density (Zn redox kinetics) can reach the maximum value in a concentration range of 6–7 m KOH. Even though the Zn^2+^ ions can be reduced to metal Zn during the charging process. However, the Zn^2+^ in the alkaline electrolyte can also further react with OH^−^, forming a passivation layer and further making it difficult to reduce Zn^2+^ to metallic state. When the pH is lower than 13, passivation of Zn gives rise to the formation of stable, compact, and insoluble semiconductor ZnO (Equation (2) and Figure 2c), which is inconvenient for ZABs operation. In addition, as the increased ZnO solubility with KOH concentration, Zn^2+^ will form, migrate, and redeposit back to Zn anode unevenly, exacerbating the dendrite nucleation growth, shape reconstruction, and surface passivation of the Zn anode.

### 3.2 HER on the Zn Anode

The standard reduction potential of Zn^2+^ to Zn is −1.25 V (vs standard hydrogen electrode [SHE], at pH = 14). While the standard potential for HER is −0.83 V (vs SHE, Equation (4)), meaning that the HER is thermodynamically favored under the ZABs operation conditions, leading to the corrosion of the Zn anode (Equation (5)), self‐discharge (Figure 3a), and reduced CE. In 6 m KOH, the HER on the Zn anode possesses a Tafel slope of 124 mV dec^−1^ and an exchange current density of 9.5 × 10^−7^ mA cm^−2^.^[^ ^18^ ^]^ The HER current density on the Zn surface is ≈10^−5^ mA cm^−2^ at the standard reduction potential of Zn/Zn^2+^.^6^, ^18^ More seriously, the HER overpotential on the ZnO surface will be significantly reduced,^[^ ^19^ ^]^ signifying that the self‐discharge rate of the Zn anode will increase with the formation of ZnO during discharging (Equation (6)). Efficient strategies and methods to suppress the HER are needed to improve the CE and inhibit the self‐discharge of ZABs. The dendrite growth, shape reconstruction, and surface passivation of the Zn anode, as well as HER, will interact and be entangled with each other on the Zn anode.

(4) $$H_{2}O+e^{-}\to 2{\text{OH}}^{-}+H_{2}(g)\;(E^{0}=-0.83\text{ V vs SHE})$$

(5) $$\text{Zn}+2H_{2}O\to \text{Zn}{\left(\text{OH}\right)}_{2}+H_{2}(g)$$

(6) $$\text{Zn}{\left(\text{OH}\right)}_{2}\to \text{ZnO}+H_{2}O$$

### 3.3 Strategies for Alleviating the Surface Side Reactions

In terms of cell design engineering for ZABs, Zn–air flow cells can be considered to reduce the surface side reactions with the help of a flowing system compared with the stationary ZABs.^6^, ^20^ In the ZABs flow cells, recycling the fresh anode alkaline solution (dispersed metallic Zn slurry) or the fresh alkaline aqueous electrolyte to the reactor is an efficient way to alleviate the surface side reactions. The flowing system will not only help improve the discharge/charge efficiency of the Zn anode but also avoid the formation of the by‐product from side reactions, thus achieving favorable O_2_ access due to less cathode clogging. Also, the structure of ZABs flow cells with the flowing electrolyte can inhibit the Zn dendrite growth effectively compared with the stationary ZABs without the flowing system. Consequently, the dendrite‐free morphology of the Zn anode was observed in the flowing electrolyte instead of the stationary electrolyte.^[^ ^21^ ^]^ Thus, the ZABs flow cells exhibit unparalleled battery performance, including superior power output and excellent cycling stability. Some representative works about ZABs flow cells showing significantly improved battery performance, stability, and efficiency than the stationary ZABs have been reported recently.^6^, ^20^, ^22^ However, the ZABs flow cells need the supernumerary tubes, pumps, and excessive electrolyte, increasing the system complexity and cost. Also, the external power pump is necessary for the ZABs flow cells to drive the liquid flow, which may consume extra energy.^6^, ^20^

In addition, from the electrochemical and material science aspects, other strategies including increasing surface area, fabricating 3D electrode structure, adding additives to the electrodes/electrolytes, and electrode coating are also used to improve the ZABs cyclability.^11^, ^23^ The increased surface area of the Zn anodes will facilitate the surface reaction kinetics, thus mitigating the Zn dendrite formation during the charging process. It should be noted that with the increased surface area of the Zn anode, the reaction rate of HER will also increase due to the exposure of active sites. To improve the mechanical stability and alleviate the shape reconstruction of the Zn anode, the polymeric binders, such as polytetrafluoroethylene (PTFE), ^[^ ^24^ ^]^ carboxymethyl cellulose (CMC),^11^, ^24^ agar,^[^ 25, 26 ^]^ and poly(vinylidene ﬂuoride) (PVDF),^[^ 26, 27 ^]^ are added to the anodes. The binders can make much better dispersion of Zn and ZnO powders, which inhibits the dendrite growth of Zn and increases the effective surface area. In addition, due to the good conductivity and chemical stability in the alkaline environment, some carbon‐based additives (i.e., carbon black) are used on the Zn anode to improve the utilization efficiency of Zn and avoid surface passivation. The use of heavy metals (i.e., Bi, In, Pb, Cd, Ti, Sn, etc.) and their compounds (oxide, hydroxides, and nitrides) additives^[^ ^28^ ^]^ is also an effective route to improve the conductivity and current distribution of the Zn anode. The heavy metals can maintain their metallic phases, while the active Zn has been discharged and converted to insulating ZnO. These heavy metals usually possess much higher overpotentials than Zn/ZnO for HER, thus inhibiting the various surface side reactions simultaneously. The HER on the Zn electrode can also be alleviated by reducing the H_2_O activity using a highly concentrated Zn‐ion electrolyte (HCZI),^[^ ^29^ ^]^ in which a unique solvation‐sheath structure of Zn^2+^ will be formed in the HCZI electrolyte, making the (Zn‐TFSI)^+^ much easier to be formed due to the huge amounts of anions forces and thus significantly suppressing the formation of (Zn‐(H_2_O)_6_)^2+^. Therefore, the HCSI not only retains water in the open atmosphere, but also enables dendrite‐free Zn plating/stripping at nearly 100% CE. Moreover, using a polymer electrolyte can further inhibit the HER on Zn electrode.^[^ 27, 30 ^]^

The dissolution and deposition of Zn and ZnO can be tuned by optimizing the structure of the Zn anode, thus minimizing the shape reconstruction of the Zn anode during long‐term operation. For example, Debra R. Rolison reported the use of a 3D Zn sponge anode in alkaline Zn batteries (Figure 3c).^[^ ^31^ ^]^ The chemical reactions of Zn in the nickel‐3D zinc batteries are similar to those in the ZABs. The developed 3D Zn sponge anode showed minimal shape reconstruction and no dendrite formation was observed, while severe dendrite was found to form on traditional Zn powder anode. A much higher utilization efficiency of Zn was obtained due to the monolithic structure and improved surface area of the 3D Zn sponge structure. Due to the novel 3D sponge structure, the well‐distributed current and electrolyte, as well as superior performance, were obtained. Almost 90% of the theoretical capacity was achieved in the charging process, and more than 95% of the capacity can be recovered. However, as the increased surface area and reduced electrical conductivity of the 3D Zn sponge anode, the HER should be carefully addressed as we discussed earlier.

Other strategies, such as electrolyte additives (such as K_2_CO_3_, KF, K_3_PO_4_, and K_3_BO_3_), electrode coating with other materials have also been investigated.^[^ ^32^ ^]^ The coating method should allow sufficient migration of OH^−^ ions to facilitate the charge and discharge processes, but simultaneously reduce the migration rate of Zn(OH)_4_^2−^ outward during the discharge process. The shape change in the Zn electrode can be mitigated and the concentration gradients can be reduced due to the inhibited Zn(OH)_4_^2−^ migration during the charging process, which lowers the driving force for dendritic growth and significantly improves the cycle life of ZABs.^[6a]^ For example, the Zn metal surface coated with Li_2_O‐2B_2_O_3_ will not only suppress the HER but also increase the discharge capacity due to that the coating layer prevents the direct contact of the Zn surface with the strong alkaline electrolyte, thus avoiding the side reactions of the Zn electrode.^[^ ^33^ ^]^ The corrosion resistance can be increased by coating with neodymium conversion films on the Zn electrode and thus stabilizing the cycle behavior of the Zn electrode.^[32c]^ Recently, a porous nano‐CaCO_3_ coating layer as a buffer layer which can lead the uniform and position‐selected Zn stripping/plating on Zn foil interfaces was reported (Figure 3d),^[^ ^34^ ^]^ The high porosity of CaCO_3_ can be readily permeated by the aqueous electrolyte, guiding a relatively uniform electrolyte ﬂux and Zn plating rate over the Zn foil surface. The small‐sized Zn nuclei would be confined in the nanopores of the CaCO_3_ coatings, lowering polarizations of these electrodes. In addition, a large potential variation across the nano‐CaCO_3_ coating was existed due to its electrically insulating nature. Therefore, only the potential near the Zn foil surface was negative enough for Zn^2+^ reduction, leading to a position‐selected, and dendrite‐free stripping/plating process, which delivers a 42.7% higher discharge capacity than bare Zn electrode even after 1000 cycles. It should be noted that the coating strategy, including inorganic and organic coating layers, is still limited in improving the performance and life of the Zn anode. The inorganic coating layer is brittle, which is easy to fracture under a long‐time cycle and fast Zn plating/striping. The organic coating layer is flexible; however, the hydrophobic polymer layer will cause a drastic increase in polarization potential for Zn plating/stripping owing to the elevated nucleation barrier and restricted 2D diffusion of Zn.^[^ ^35^ ^]^

In addition to the coating method, another effective method to inhibit the dendrite growth and surface passivation is controlling the interfacial electrochemistry on the Zn/electrolyte interface.^[^ ^36^ ^]^ The combination of the inorganic and organic composite coating layer (Nafion–Zn–X) method was also developed to take the advantage of coating and interface electrochemistry.^[^ ^35^ ^]^ The composite protection layer formed in the interface of Nafion–Zn–X can not only shield anion and free H_2_O to suppress the side reactions, but also restrain the Zn dendrites by uniform Zn plating/stripping. A composite protective layer consisting of nanosized metal–organic frameworks (MOFs) and PVDF was also developed to improve the poor wetting effect of aqueous electrolytes on the Zn anode to reconstruct the Zn/electrolyte interface.^[^ ^37^ ^]^ In the MOF–PVDF layer, the hydrophilic MOF nanoparticles serve as interconnecting electrolyte reservoirs, enabling nanolevel wetting effect as well as regulating an electrolyte flux on Zn anode. This zincophilic interface exhibits significantly reduced charge‐transfer resistance. Thus, stable and dendrite‐free Zn plating/stripping cycling performance is achieved for over 500 cycles.

**Figure 4:**
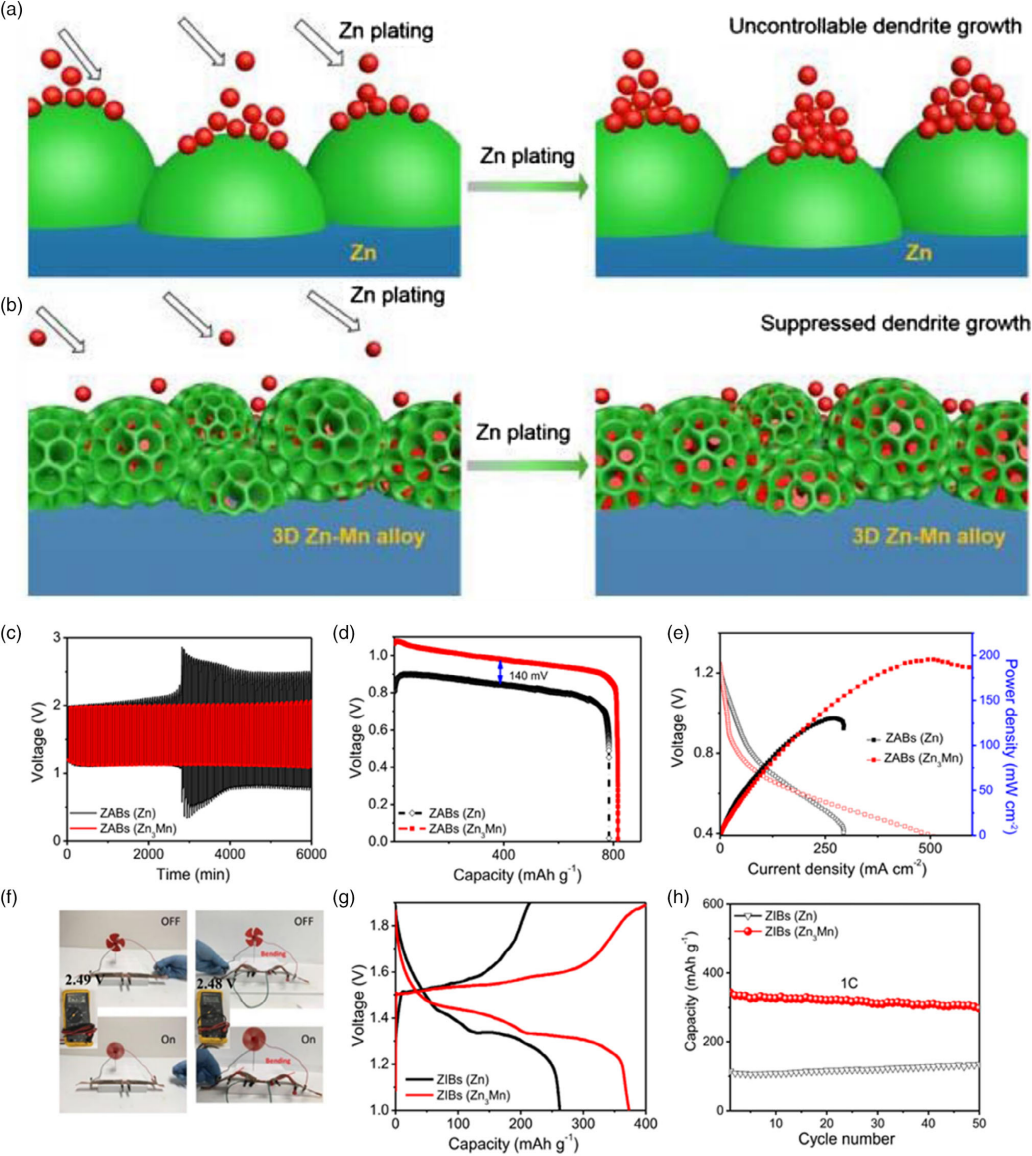
Typical Zn plating processes on a) Zn anode and b) Zn_3_Mn alloy anode. c–h) Electrochemical performance of aqueous Zn batteries. Reproduced with permission.^[11b]^ Copyright 2021, Springer Nature.

Recently, an electrodeposition approach was developed to fabricate a 3D Zn_3_Mn alloy anode to address the Zn dendritic growth and interfacial instability issues, which was proved to significantly improve the electrochemical performance of ZABs.^[11b]^ As the more negative reduction potential of Mn/Mn^2+^ than Zn/Zn^2+^, the electrostatic shield effect can help inhibit the Zn dendrite formation during the Zn deposition on the Zn—Mn alloy surface. Compared with the traditional Zn plate with a dendrite growth, the Zn_3_Mn anode surface with a homogeneous Zn coverage was achieved because that the proper bonding between Zn and Mn in the Zn_3_Mn alloy provides fast Zn diffusion channels and therefore suppresses dendrite growth (**Figure** **4**a). The in‐depth study indicates that both the reaction kinetics and thermodynamics on the 3D Zn_3_Mn electrode can be well controlled. The minimal dendrite formation, Zn nucleation growth at the initial stage of the plating can be guided and regulated by the electronic structure of the Zn_3_Mn alloy. As a proof of concept, the as‐developed Zn_3_Mn alloy showed much better cycling performance, discharge capacity, and power density than the ZABs using commercial Zn anodes (Figure 4c–e). Also, it can work as flexible ZABs (Figure 4f–h), showing outstanding charge/discharge and cycling performance for ZABs.

**Figure 5:**
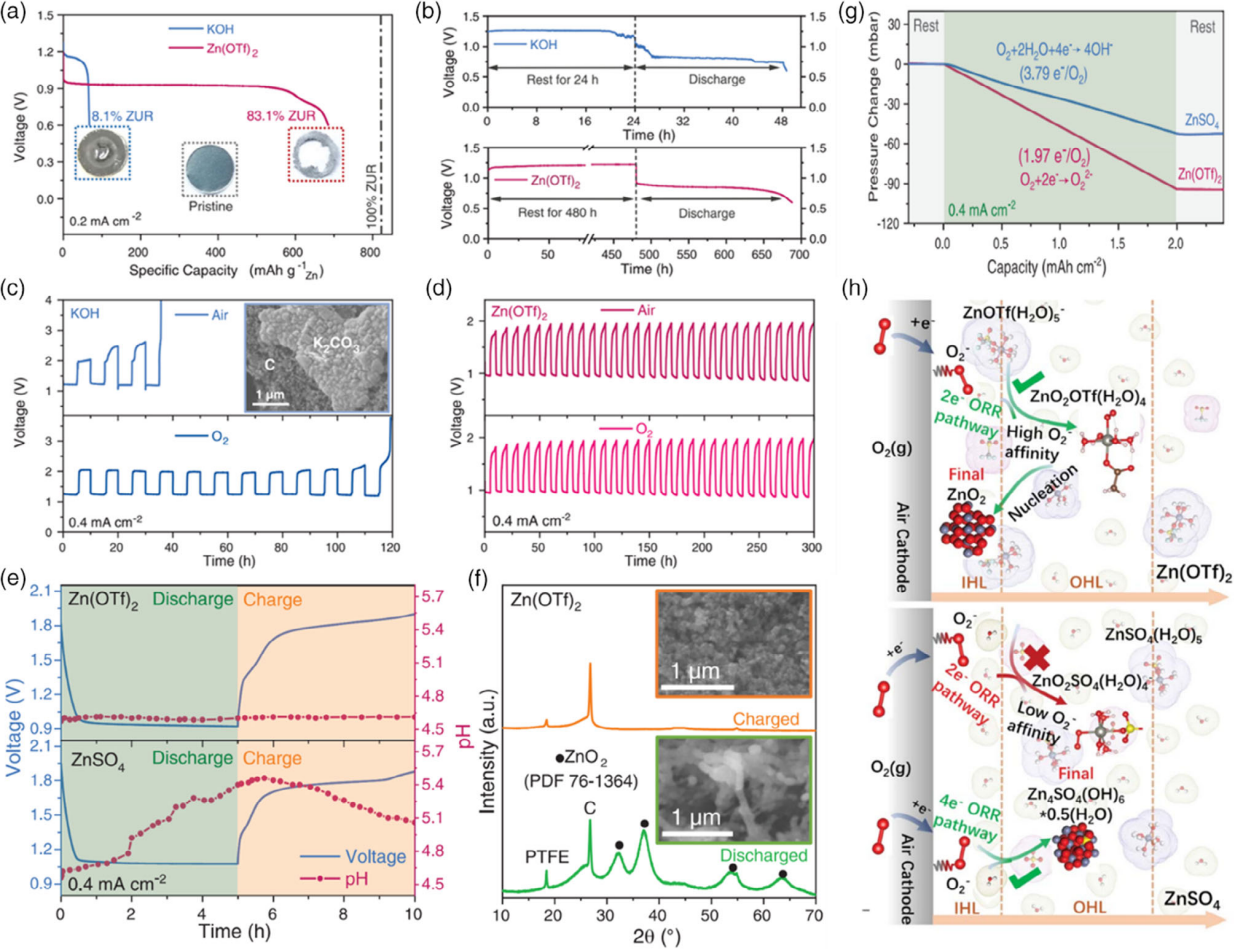
a–d) Comparison of the electrochemical performance in a Zn(OTf)_2_ electrolyte and KOH electrolyte. e) The galvanostatic discharge and charge profiles of the batteries using Zn(OTf)_2_ and ZnSO_4_ electrolytes and the corresponding recorded electrolyte pH values. f) X‐ray diffraction (XRD) and scanning electron microscopy (SEM) of air cathodes after discharge and recharge in Zn(OTf)_2_ electrolytes. g) Pressure change in Zn—O_2_ cells using Zn(OTf)_2_ and ZnSO_4_ electrolytes during a discharge process under pure O_2_. h) The reaction processes in the inner Helmholtz layer (IHL) and outer Helmholtz layer (OHL) on the air electrode surface in different electrolytes, respectively. Reproduced with permission.^[^ ^90^ ^]^ Copyright 2021, American Association for the Advancement of Science.

Generally, the supersaturated Zn(OH)_4_^2−^ will be decomposed to compact and insoluble semiconducting ZnO, which decreases the performance of ZABs (Figure 2c). Recently, a Zn peroxide (ZnO_2_) was proposed through a 2e^−^/O_2_ process in nonalkaline aqueous electrolytes. In contrast to the traditional used alkaline solution, the hydrophobic trifluoromethanesulfonate (OTf^−^) anion with a large molecule size was used as electrolyte solute, and the so‐called Zn^2+^‐rich and water‐poor inner Helmholtz layer was established for the air cathode, thus promoting the aprotic 2e^−^ ORR (**Figure** **5**). Compared with the widely used KOH electrolyte, the adopted OTf^−^ anions‐based electrolyte significantly increases the Zn utilization ratio (ZUR) by more than ten times (83.1% vs 8.1%, Figure 5a). In addition, compared with the traditional KOH electrolyte in ZABs which was susceptible to the pH change and CO_2_ contamination from the atmosphere, the OTf^−^ electrolyte‐based ZABs show more sustainable and stable performance in air and no obvious change in pH during the charge/discharge cycling (Figure 5b–e). The ZnO_2_/Zn has high reversibility during the charge/discharge operation (Figure 5f). Different from the traditional aqueous KOH electrolyte with a 4e^−^ pathway, a 2e^−^ chemistry was obtained in this OTf^−^ electrolyte (Figure 5g). The Zn^2+^‐rich, H_2_O‐poor structure, and the weak attraction between Zn^2+^ cations and OTf^−^ anions are the two major reasons to promote 2e^−^ ORR chemistry (Figure 5h). In future research, the ionic conductivity and viscosity of OTf^−^ should be considered to improve the rate performance and power density of ZABs.

## 4 Chemistry and Materials of the Air Cathode

When discharging ZABs, the O_2_ from the atmosphere diffuses into the air cathode and is reduced to oxygen‐containing species (OH^−^, Equation (3)) through ORR. For the primary ZABs, the consumed Zn anode must be mechanically replaced with a new Zn anode. With the development of ZABs, the rechargeable ZABs attracted much attention to reducing the cost of ZABs. The discharging process in the rechargeable ZABs will provide electricity, while transient energy such as wind energy and solar energy can be used to recharge the ZABs. Especially, the rechargeable characteristic is vital for electric vehicles. During the charging, the electrochemical process is the reverse reaction of Equation (3) at the air cathode, in which the OER will proceed.

The performance of electrically rechargeable ZABs largely relies on the air cathode with bifunctional activity and durability, which will face harsh conditions during the repeated discharge/charge processes in aqueous alkaline electrolytes. Developing bifunctional ORR/OER catalysts is a great challenge due to the large overpotential between the ORR and OER processes. The materials for air cathodes should be both chemically and electrochemically stable in a strongly alkaline solution. The electrocatalysts for air cathodes must keep high activity for ORR/OER under both highly reductive and oxidative environments under high current densities. A wide voltage window from 0.5 to 2.0 V (pH = 14) is usually used in ZABs. Some reports are advocating that a three‐electrode system (where the Zn anode is located between the ORR electrode and OER electrode) can be used to replace the two‐electrode system,^8^, ^38^ in which the ORR and OER occur during the discharge and charge process, respectively. For the three‐electrode system, the exposure risk of ORR electrocatalysts (or OER electrocatalysts) to the oxidative (or reductive) environments is avoided, and therefore monofunctional ORR and OER catalysts can be used, which will dramatically improve the batteries lifetime as well as the cycling stability. However, because of the relatively complicated cell design of the three‐electrode ZABs, two‐electrode ZBAs using bifunctional catalysts on the air cathodes are still dominating the research in the field. The proposed three‐electrode system can be used for electrocatalysts assessment at the start‐up stage, optimizing and enhancing the battery cycling stability.

### 4.1 Air Cathode Structure

**Figure 6:**
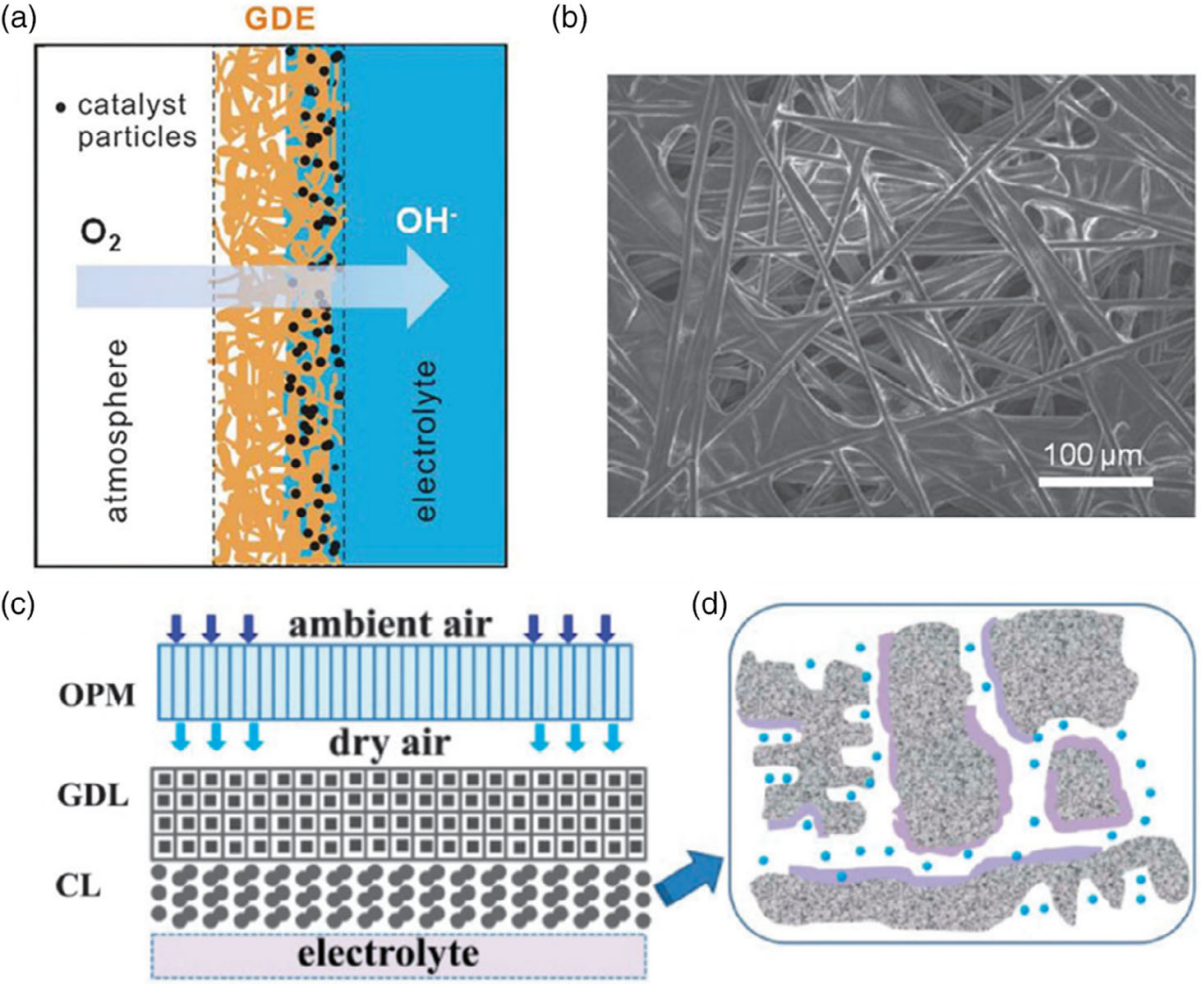
a) Catalysts supported on the gas diffusion electrode in the liquid electrolyte. b) SEM image of the widely used commercial Toray carbon paper. The carbon paper was treated with hydrophobic PTFE to enhance the water‐proofing. Reproduced with permission.^[7b]^ Copyright 2014, Royal Society of Chemistry. c) Air cathodes with metal mesh (foam)‐supported oxygen permeable membrane (OPM) to eliminate H_2_O or CO_2_. d) A CL with hierarchical pores. Reproduced with permission.^[7a]^ Copyright 2012, Royal Society of Chemistry.

The ORR/OER takes place at a gas–liquid–solid (gaseous O_2_, liquid KOH electrolyte, and solid catalysts) three‐phase interface. Therefore, a boundary with a high surface area between the three phases is essential for highly active and efficient air cathodes. During ORR, the O_2_ diffusion from the atmosphere through the gas phase is much easier and quicker than that through the liquid electrolyte because of the much lower solubility and diffusivity of O_2_ in most solvents (**Figure** **6**a). Traditional cathode structures for ZABs cannot provide enough three‐phase interface zones to meet the requirements for ORR/OER occurring under the condition of high current density and continuous operation. The porous gas diffusion electrodes were used in the early stage of ZABs development. The surface/interface of the gas diffusion electrode consists of a hydrophobic gas diffusion layer (GDL) and a moderately hydrophilic catalyst layer (CL). The GDL guarantees the smooth diffusion of O_2_ from the air into the reaction interface, while the CL supported on the GDL catalyzes the ORR/OER. The GDL not only provides gas channels for O_2_ diffusion in and out during the discharge (ORR) and charge (OER) but also serves as conductive support for the catalysts. Furthermore, the GDL also acts as a wet‐proofing layer to prevent the leakage of the aqueous electrolyte. An ideal air cathode should contain a hydrophilic CL and hydrophobic GDL. Noted that suitable humidity is crucial for the ZABs performance. Low humidity will result in the gradual evaporation and drying up of the electrolyte, while high humidity will cause the flooding of the air cathode. Therefore, the well‐balanced hydrophilic and hydrophobic properties of the air cathode are very important for ZABs. Therefore, the rational design of air cathodes with optimal hydrophilicity and friendly interface structure is crucial to ZABs. Nowadays, the most widely used commercial GDL is designed and developed by Toray Industries, Inc. (Japan). In the Toray carbon paper, the GDL with high porosity and electrical conductivity was acquired, in which the carbon fibers are tied together by graphitized carbon layers. After treated by wet‐proofing agents PTFE, the Toray carbon paper can be used for various research purposes (Figure 6b). Even though the Toray carbon paper (carbon cloth) has shown big success as GDL, especially in the proton exchange membrane (PEM) fuel cell research field, however, it should be kept in mind that the ZABs need to be operated under harsh conditions. Apart from the ORR, the OER needs a much higher working voltage (generally above 2.0 V), which is higher than the carbon oxidative corrosion voltages.^[^ ^39^ ^]^ Thus, it is urgent to develop cheaper and alternative GDL, which should possess good anticorrosion property, excellent electrical conductivity, and high surface area.

Another important issue for ZABs is the diffusion of CO_2_ and H_2_O from the atmosphere into the air cathode together with O_2_. The CO_2_ will react with the alkaline electrolyte and thus form carbonate precipitation, which can block the diffusion/transport channels. In addition, the H_2_O can also dilute the electrolyte and change the pH of the electrolyte, affecting the ZABs performance. Therefore, managing the CO_2_ and H_2_O diffusion through a selective membrane only allowing O_2_ diffusion but excluding/blocking CO_2_ and H_2_O is feasible (Figure 6c,d).^[^ ^40^ ^]^

### 4.2 Bifunctional ORR/OER Catalysts

The most efficient ORR and OER electrocatalysts have been developed using scarce and expensive platinum metal groups (PGMs) materials (i.e., Pt, Pd, IrO_2_, RuO_2_, etc). The Pt and IrO_2_ (RuO_2_) are still the benchmark catalysts for evaluating the newly developed materials. The large‐scale application of PGMs in ZABs is severely limited by their high costs. Moreover, the rechargeable ZABs require bifunctional catalysts, which can simultaneously catalyze the ORR/OER reactions during the discharge/charge processes. However, these PGMs always show deficient bifunctional activities. For example, as the state‐of‐the‐art ORR catalyst, Pt shows inferior OER performance due to the formed oxide layer with low electrical conductivity on Pt surface at OER working potential. Therefore, two different types of PGMs usually need to be used in a physical mixture to gain bifunctionality, while the electrocatalytic performance of the mixed catalysts is bound to be affected. Therefore, higher PGMs loading is needed to compensate for the activity loss of these mixed electrocatalysts, further raising the cost of PGMs as ZABs electrodes. Therefore, it is advisable to reduce the PGMs usage and use the earth‐abundant nonprecious materials as ZABs electrocatalysts.

**Figure 7:**
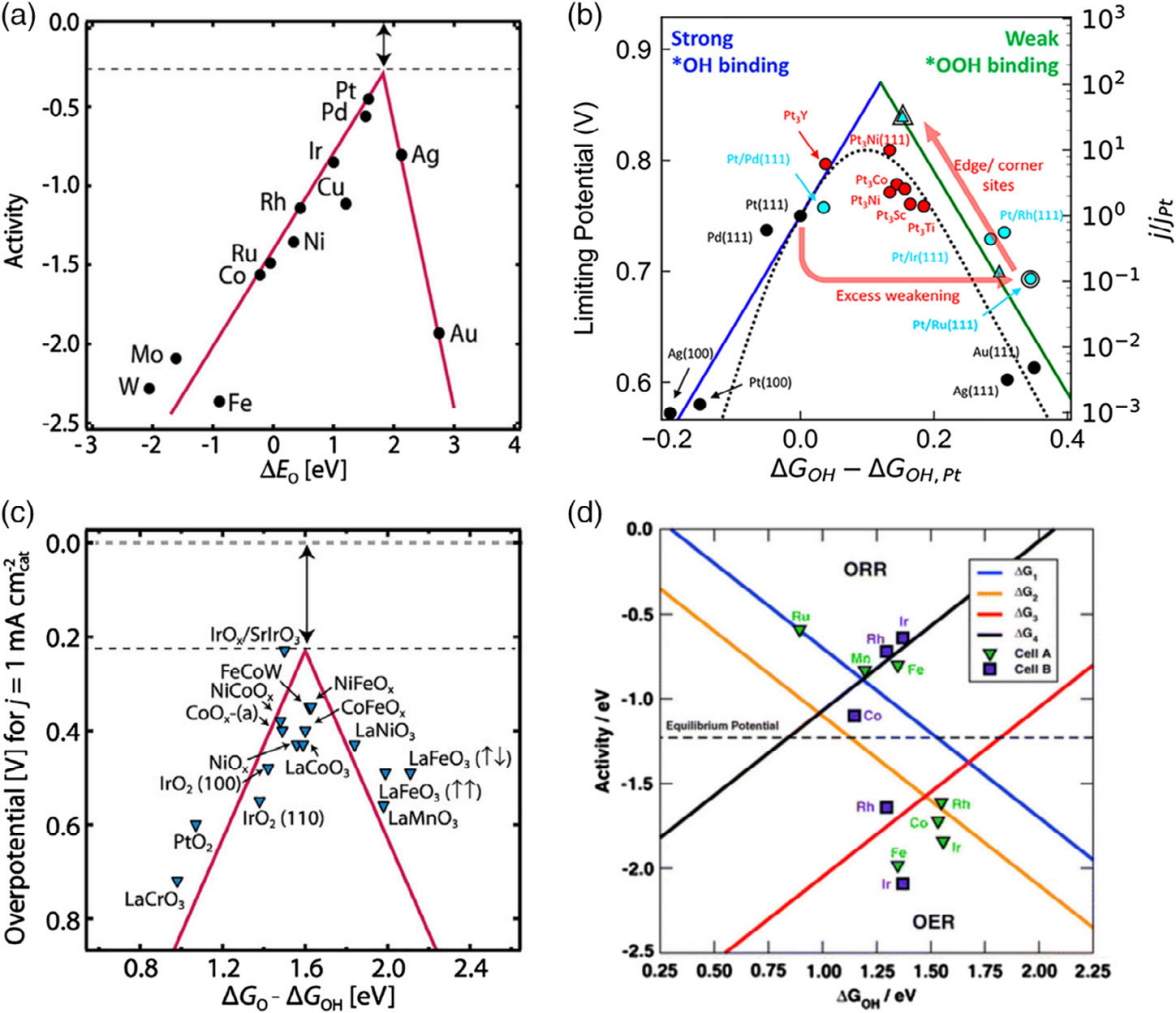
a) ORR volcano plot for different metals. Reproduced with permission.^[^ ^99^ ^]^ Copyright 2014, American Chemical Society. b) Kinetic volcano and the limiting potential volcano for typical metals and alloys. Reproduced with permission.^[^ ^41^ ^]^ Copyright 2018, American Chemical Society. c) OER volcano plot for metal oxides. Reproduced with permission.^[1c]^ Copyright 2017, American Association for the Advancement of Science. d) Double volcano for OER and ORR. Reproduced with permission.^[^ ^100^ ^]^ Copyright 2011, Royal Society of Chemistry.

At the early stage of ZABs development, the PGMs were first used and studied to lower the overpotential and increase ZABs efficiency. In general, the four elementary reactions for ORR can be expressed as follows (Equation ((7), (8), (9))–(10), reversed processes for OER).

(7) $$*+O_{2}+H_{2}O+e^{-}\to *\text{OOH}+{\text{OH}}^{-}$$

(8) $$*\text{OOH}+e^{-}\to *O+{\text{OH}}^{-}$$

(9) $$*O+H_{2}O+e^{-}\to *\text{OH}+{\text{OH}}^{-}$$

(10) $$*\text{OH}+e^{-}\to *+{\text{OH}}^{-}$$

where * represents the active site for ORR. At least three oxygen‐containing intermediates (i.e., *OOH, *O, and *OH) are involved in the four‐electron transfer ORR processes in alkaline solution. Theoretically, the volcano plots (**Figure** **7**) have been widely used to present the scaling relations of the binding energies of the reactants on different catalytic surfaces. Both the theoretical and experimental results indicate that Pt, Pd, and Ag are among the best metals for ORR, sitting almost on the apex of the volcano (Figure 7a). However, even for the most reactive catalyst with the optimal adsorption strength at the peak of the volcano plot, an overpotential of 0.3–0.4 V was still found (Figure 7a). Based on the scaling relation, two effective strategies can be used to further boost the ORR activity (Figure 7b), including tunning the d‐band electronic structure (strain, surface orientation, and composition) and regulating the intermediates’ adsorption sites (coordination environments).^[^ ^41^ ^]^

Due to the high charging voltage (usually around 2 V), most of the OER catalysts are metal oxides (Figure 7c). The OER also goes through a four‐electron process and has a general adsorption‐energy scaling relationship on the surface of oxides, including spinel, perovskite, bixbyite oxides, and so on. Like ORR, the best OER catalyst still shows overpotentials from the scaling relation (Figure 7c). The OER performance can be further boosted through various strategies, such as morphology fine‐tuning, crystal facet control, composition regulation, defect, strain, and doping engineering.^[^ ^42^ ^]^ For example, the hierarchical mesoporous Co_3_O_4_ nanowire array possesses much higher bifunctional OER/ORR activity than other morphologies.^[^ ^43^ ^]^ The α‐MnO_2_ with nanospheres and nanowires morphologies has a much better OER performance than that with microparticles morphology due to the smaller particle size and higher specific areas.^[^ ^44^ ^]^ The Co_3_O_4_ with exposed (111) planes has a much higher OER performance than that with exposed (001) planes due to the much lower O_2_ desorption activation barrier.^[^ ^45^ ^]^ Regulating the elemental composition of A‐site or B‐site in perovskite (ABO_3_ structure) also can boost the OER performance due to the partial reduction of Ni^3+^ to Ni^2+^ as a result of oxygen vacancies (OVs).^[^ ^46^ ^]^ The bandgap will be narrowed on account of the donors of the OVs, thus increasing the electron density and electrical conductivity of materials. In addition, the surface of OVs can result in the enhancement of the electron transfer from O‐vacancies to metal d‐band, thus tuning the adsorption of surface species for OER/OER.^[^ ^47^ ^]^ As proved by the previous study,^[^ ^48^ ^]^ the OER activity of perovskite oxide has a volcano relationship with the numbers of e_g_ electron. Thus, the degree of e_g_ orbital splitting and polarization can be adjusted by the strain engineering.^[^ ^49^ ^]^ The heteroatom doping (N, P, S, B, and so on) into carbon results in multiple possible configurations, making the neighboring carbon atoms electron‐deficient, thus contributing to the oxygen adsorption.^[^ ^50^ ^]^ Although great efforts have been made for the development of ORR and OER electrocatalysts, the bifunctional catalysts are inherently not the best option due to the separated vertices of the ORR/OER volcano plots (Figure 7d). However, due to the essential role of the bifunctional catalyst, both the OER and ORR linear scaling relation derived from volcano plots should be considered in the future (Figure 7d). We will summarize the bifunctional catalysts as follows.

#### 4.2.1 PGMs and Their Alloys

PGMs such as Pt have been widely studied as air cathodes in ZABs. The Pt catalysts anchored in high surface area carbon (Pt/C) were physically mixed with OER catalyst IrO_2_ (RuO_2_) and used as standard air cathodes for ZABs. The scarcity and high cost of these PGMs urge the researchers to develop effective approaches to maximize its activity and stability with reduced loading,^[^ ^51^ ^]^ such as reducing the dimensions of Pt down to nano or even atomic scales and tunning the morphology and crystal facets.^[^ ^52^ ^]^ Another effective and viable strategy is alloying Pt with other less‐expensive noble metals (such as Pd),^[^ ^53^ ^]^ heavy metals (such as Pb),^[^ ^54^ ^]^ and transition metals (such as Fe, Ni, Co, Mo, and Cu).^[^ ^55^ ^]^ Recent studies indicate that the Pt_3_Ni (111) alloy possesses an uncommon electronic structure, where the highly structured compositional oscillation was found in the near‐surface region. It is found that the outermost and third layers of Pt_3_Ni (111) are Pt‐rich, while the second layer is Ni‐rich.^[55e]^ The Pt_3_Ni (111) surface exhibits more than ten times higher ORR activity than the Pt (111) surface. Also, a relationship between the surface composition, electronic structure, and the ORR‐specific activity of Pt_3_M (M = Fe, Co, Ni, V, and Ti) was established by Markovic.^[^ ^56^ ^]^ They found that the Pt_3_Co and Pt_3_Ni possessed the best ORR performance, which is in line with the results shown in Figure 7b.

**Figure 8:**
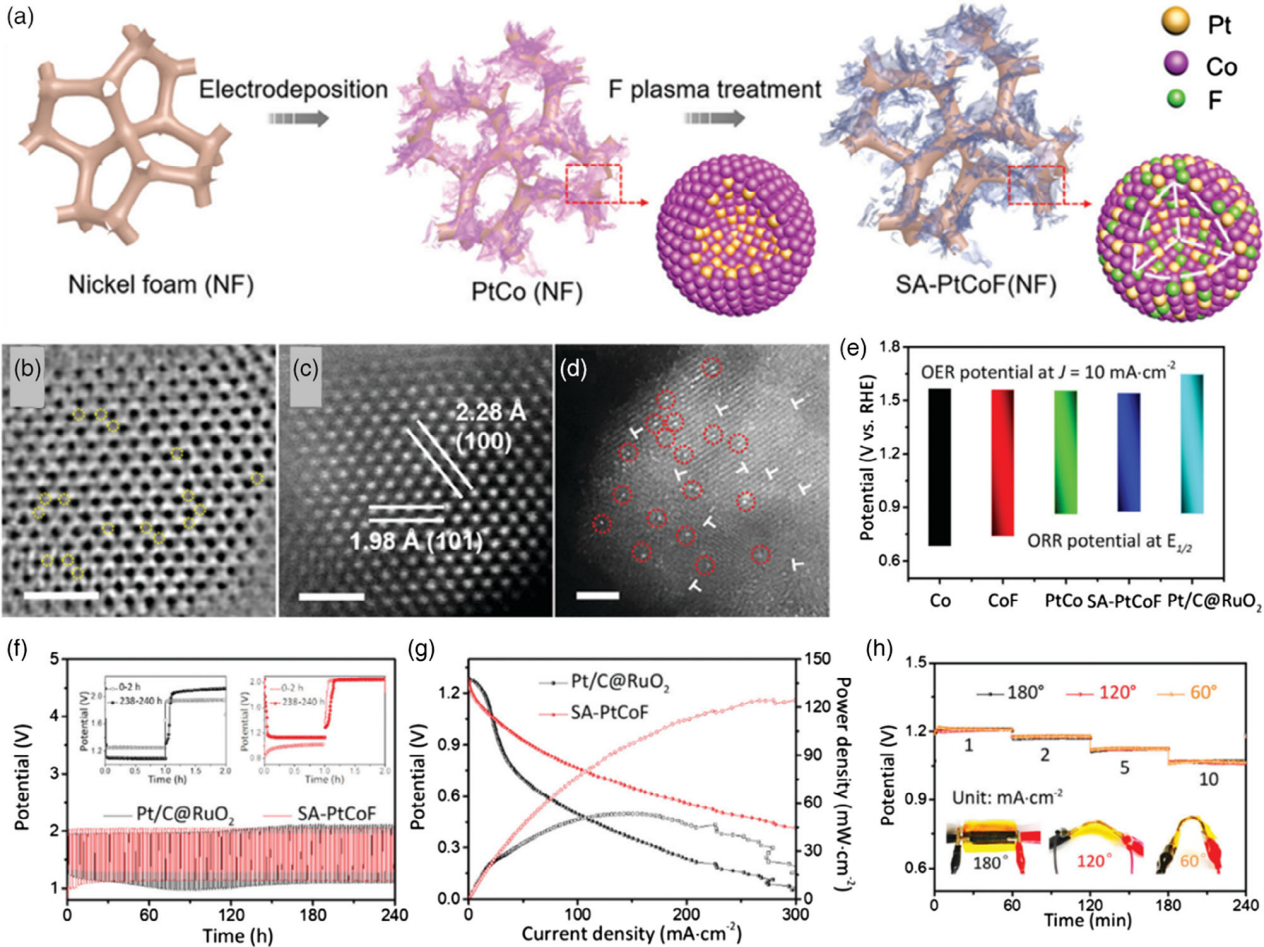
a) Schematic fabrication for the synthesis of SA‐PtCoF nanosheets. b–d) Annular bright field‐scanning transmission electron microscopy (ABF‐STEM) and high‐angle annular dark‐field (HAADF)‐STEM images. e) Overall bifunctional ORR/OER performances. f–h) ZABs performance of SA‐PtCoF. Reproduced with permission.^[55g]^ Copyright 2020, Royal Society of Chemistry.

Some significant progresses in Pt (Pd)‐based materials have been made for ORR in hydrogen–oxygen fuel cells, while it is rarely reported for OER due to the formation of surface oxide layer during charging process under high voltage. Recently, a novel method to immobilize Pt atoms in platinum cobalt (PtCo) alloy nanosheets was developed by combining electrochemical deposition with fluorine‐plasma etching treatment (**Figure** **8**a).^[55g]^ The atomic Pt was stably anchoring on the surface of PtCoF due to the lattice distortion induced by interstitial F doping (Figure 8b–d). The suggested SA‐PtCoF nanosheets were directly grown on Ni foam, which is additive‐free and carbon‐free, and thus provides affluent active sites and large surface area for electrochemical reactions. The SA‐PtCoF shows excellent bifunctional ORR/OER performance (Figure 8e) and thus can be used as an efficient air cathode for ZABs, delivering a maximum power density of 125 mW cm^−2^. In addition, the ZAB exhibits a specific capacity of 808 mAh g^−1^ and excellent cyclability for over 240 h without obvious performance decay (Figure 8f–g). Due to the flexible nature of the SA‐PtCoF, there is almost no discharge voltage loss observed even under 180° bending tests (Figure 8h). Thus, this work provides an effective method to prepare carbon‐free, additive‐free, low‐Pt loading air cathodes for ZABs.

**Figure 9:**
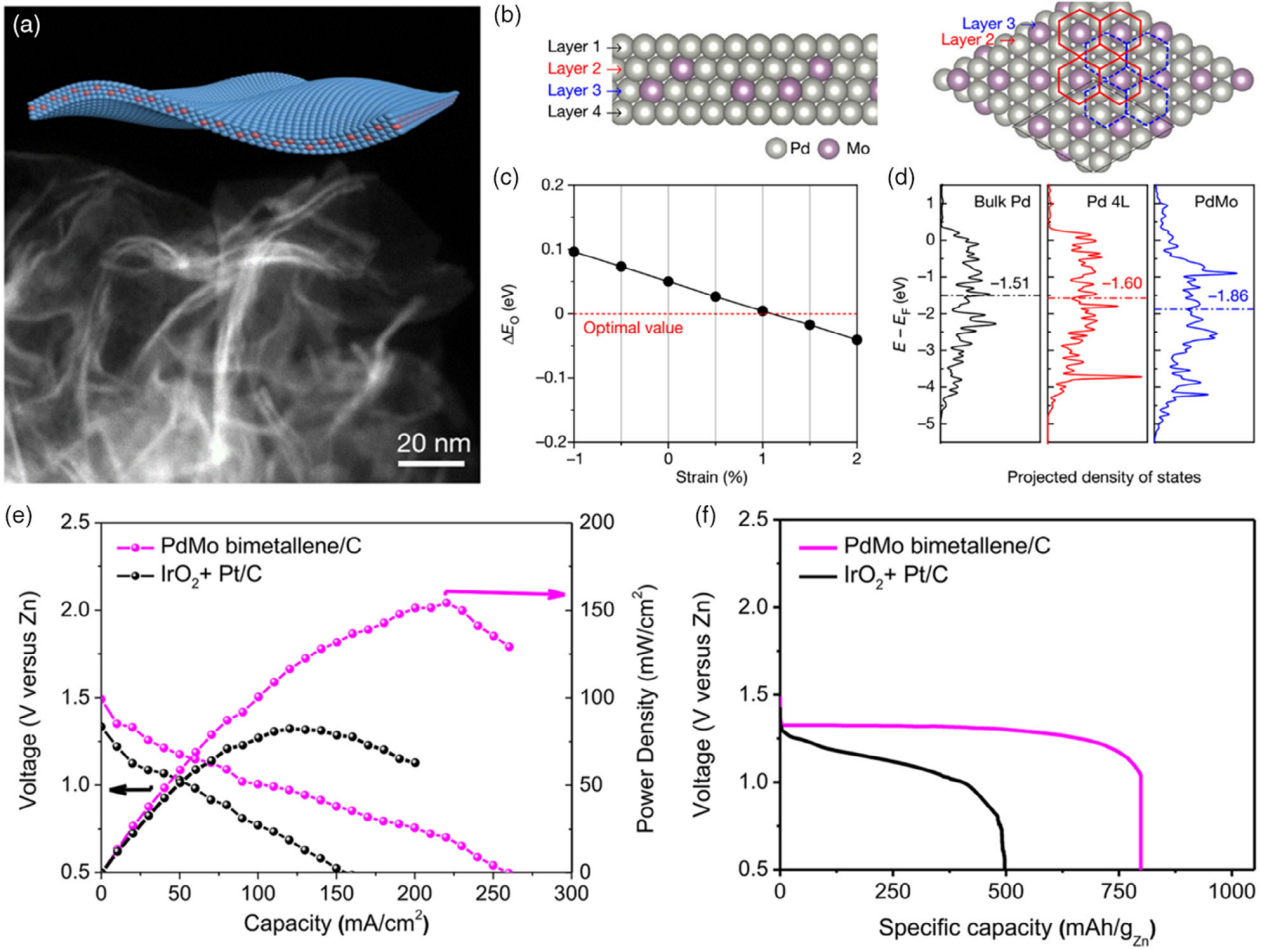
a) TEM image of PdMo bimetallene. b–d) Oxygen adsorption energy and d‐band centers of PdMn bimetallene calculated from DFT. e–f) ZABs performance of PdMo bimetallene/C compared with IrO_2_ + Pt/C. Reproduced with permission.^[55f]^ Copyright 2019, Springer Nature.

The optimization of surface strain and coordination environment in the alloys also plays a vital role in boosting the bifunctional ORR/OER performance.^[^ ^57^ ^]^ Recently, Guo's group reported superthin PdMo bimetallene nanosheets (**Figure** **9**a).^[55f]^ As Mo has a larger atomic radius than Pd, this PdMo bimetallene has a higher lattice parameter than Pd. A tensile strain of 0.95–1.4% was obtained due to the presence of Mo and the curved geometry (Figure 9a). density functional theory (DFT) calculation indicates that the optimal oxygen adsorption energy (Δ*E*_O_) can be obtained when the tensile strain is ≈1% (Figure 9b,c). The d‐band of the surface Pd atoms will be filled and shift toward negative energy with charge transfer from Mo to Pd surface (Figure 9d), driving the ORR and OER. The outstanding OER activity was attributed to the quantum size, strain, and alloying effects of PdMo bimetallene. To endow the material with good dispersion and good electronic conductivity, the PdMo bimetallene was physically mixed with carbon black and then used as ORR/OER catalysts. Impressively, the electrochemically active surface area (ECSA) is as high as 138.7 m^2^ g^−1^, and an ORR half‐wave potential of 0.95 V was obtained. The mass activity for ORR is 16.37 A mg^−1^_Pd_ at 0.9 V versus RHE in alkaline electrolytes, which almost shows the best ORR performance. In addition, the PdMo‐based ZABs show an open‐circuit voltage of 1.483 V and deliver a peak power density of 154.2 mW cm^−2^, specific capacity and energy density of 798 mAh g^−1^_Zn_ and 1043 Wh kg^−1^_Zn_, respectively (Figure 9e,f).

Other Pt‐, Pd‐, and Ag‐based materials were also in‐depth studied and reported as air cathodes over the last 4 years.^[^ ^58^ ^]^ However, bearing in mind that the high price and scarcity of these PGMs severely hinder their large‐scale applications in ZABs. The development of PGMs‐free catalysts is necessary and will promote the commercial application of rechargeable ZABs.

#### 4.2.2 Carbon‐Supported Inexpensive Metals and Alloys

The ORR/OER processes are more favorable to occur in the alkaline environments, showing faster kinetics, lower overpotentials, and much higher exchange current density than those in the acidic environments.^[^ ^59^ ^]^ According to the Nernst equation, the electrode potential will shift negatively about 0.83 V if the electrolyte changes from acid (pH = 0) to alkaline (pH = 14). The negatively shifted potential has a strong effect on the electric field at the interface between electrode and electrolyte, thus forming weak binding strengths between adsorbates and charged species.^[^ ^60^ ^]^ Thereby, the electrocatalysts show greatly weakened ions adsorption in alkaline media than in the acid, and exhibit more facile electrocatalytic processes in alkaline solutions. The much better ORR/OER performance in alkaline than in acid solution make it possible that using the non‐PGMs as bifunctional electrocatalysts for ORR/OER. Due to the low price, high surface area, and good electrical conductivity of carbon materials, carbon‐supported electrocatalysts are the most widely used materials for ORR/OER. We will introduce the most developed carbon‐supported inexpensive metals and alloys as efficient ORR/OER bifunctional electrocatalysts as follows.

**Figure 10:**
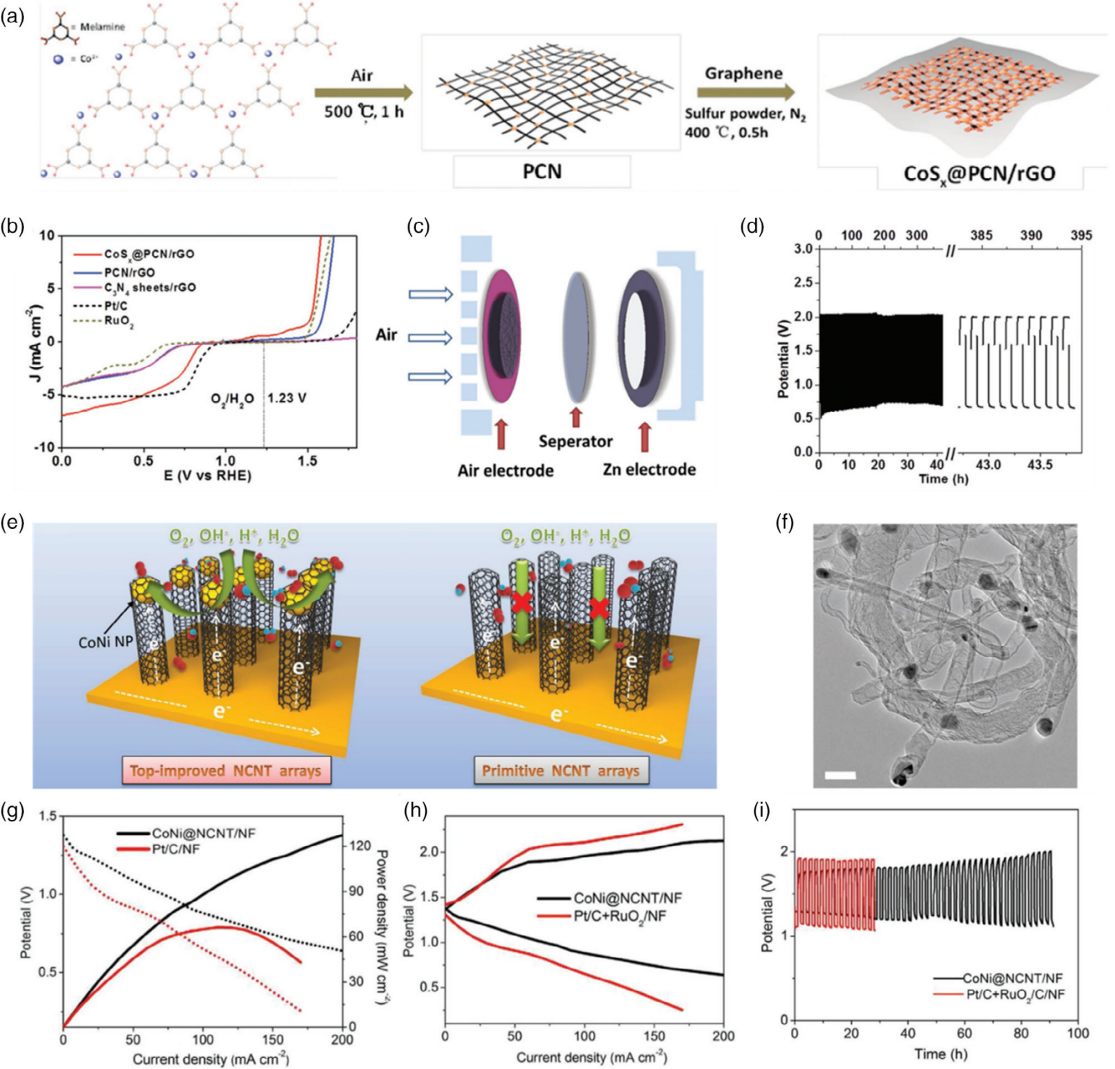
a) The preparation process of CoS_*x*_@PCN/rGO, b) ORR/OER bifunctional activity of CoS_*x*_@PCN/rGO, c) ZABs cell structure, and d) rechargeable ZABs performance of CoS_*x*_@PCN/rGO. Reproduced with permission.^[^ ^61^ ^]^ Copyright 2018, Wiley. e) Different structures and constructions of CoNi@NCNT/NF and NCNT/NF. f) TEM image of CoNi@NCNT/NF and g–i) rechargeable ZABs performance of CoNi@NCNT/NF. Reproduced with permission.^[^ ^65^ ^]^ Copyright 2018, Wiley.

The porous structure and sufficient active sites/areas of carbon‐supported catalysts are essential for electrocatalytic ORR/OER reactions. A template‐free strategy to synthesis porous carbon nitride (PCN)‐based CoS_*x*_@PCN/rGO bifunctional catalysts was recently developed.^[^ ^61^ ^]^ This template‐free and top‐down strategy can be easily controlled (**Figure** **10**a). Due to the porous structure and highly exposed active sites, the CoS_*x*_@PCN/rGO shows excellent ORR/OER bifunctional catalytic activities with the potential gap of 0.79 V between the OER (at 10 mA cm^−2^) and the ORR (half‐wave potential, Figure 10b), which is much lower than that of commercial Pt/C (1.10 V) and RuO_2_ (1.08 V). The CoS_*x*_@PCN/rGO can be used as an efficient bifunctional catalyst for ZABs (Figure 10c,d), displaying excellent cycling stability without performance decay for over 400 discharge/charge cycles. Thus, the CoS_x_@PCN/rGO shows the potential to replace PGMs as an efficient air cathode for ZABs.

The morphological emulation, such as human hair array,^[13c]^ pomegranate‐inspired structure,^[^ ^62^ ^]^ hydrangea‐like superstructure,^[^ ^63^ ^]^ and overhang‐eave carbon cages,^[^ ^64^ ^]^ also offers a way for developing efficient air cathodes. Recently, an apically dominant mechanism was proposed to improve the ZABs performance.^[^ ^65^ ^]^ In the developed catalysts, the CoNi alloy nanoparticles were encapsulated in the apical domain of nitrogen‐doped carbon (NCNT) nanotube on the Ni foam (CoNi@NCNT/NF). Compared with other alloy nanoparticles randomly loading on supports, the CoNi alloy was controlled to be mainly distributed on the top of NCNT (Figure 10e,f). In the CoNi@NCNT/NF, due to the hydrophobic nature of carbon surface, most of the ORR/OER intermediates can only approach the vertex rather than the base of NCNT arrays, which will provide ideal reaction spaces for ORR and OER. The ZABs coin cells were assembled using the CoNi@NCNT/NF as air cathodes, showing a maximum power density of 127 mW cm^−2^ and energy density of 845 Wh kg_Zn_^−1^. Furthermore, the ZABs using CoNi@NCNT/NF can operate stably for over 90 h with contineous charge/discharge cycling (Figure 10g–i).

The other nanoscale low‐cost metals^13^, ^66^ and alloys such as FeNi,^[^ ^67^ ^]^ NiCo,^14^, ^68^ FeCo,^14^, ^58^, ^67^, ^69^ as well as single‐atom metals and alloys supported on carbon^[^ 63, 64, 66, 70 ^]^ have been studied as bifunctional ORR/OER catalysts for ZABs, which show better performance than the benchmark catalysts Pt/RuO_2_(IrO_2_). In particular, the single‐atom (or isolated metal) catalysts can significantly reduce the amount as well as maximize the atomic utilization of metals, showing great potential in the development of cost‐effective ZABs. The details about single‐atom catalysts for ORR/OER and ZABs have been well summarized elsewhere.^[^ ^71^ ^]^

#### 4.2.3 Metal‐Free Carbon Catalysts

Carbon materials can exist in different forms with various electronic and electrochemical properties. Carbon atoms with *sp* ^3^ (diamond structure) and *sp* ^2^ (graphite structure) hybridization are the main existing forms. Carbon materials in a diamond structure with completely *sp* ^3^ hybridized carbon atoms have extreme hardness but low electrical conductivity, which cannot meet the requirements for electrocatalytic reactions. The *sp* ^2^ hybridized carbon materials with graphitic structure have a lot of analogs, such as graphite (such as carbon fibers, glass carbon, pyrolytic graphite, etc.), carbon nanotubes, graphene, and fullerene. The graphitic materials are suitable for electrocatalytic reactions because of their good electrical conductivity.^[7a]^ Though carbon‐supported metal (M/C) ORR electrocatalysts have been widely used, however, the pristine carbon materials without any modification display poor inherent ORR activities with a dominant 2e^−^ transfer process to form peroxides. In M/C, the carbon materials are just used as supports to promote the dispersion of the active metal, increase the electrical conductivity, and form a strong metal–support interaction to immobilize the metallic active sites.^[^ ^72^ ^]^ Thus, the metal‐free carbon materials are usually doped by other heteroatoms such as nitrogen (N), boron (B), fluorine (F), phosphorus (P), sulfur (S), and selenium (Se) to boost the ORR/OER performance.^[^ ^73^ ^]^ With the aid of the doping effect and electron transfer between carbon and heteroatoms, the electronic properties of adjacent carbon atoms in carbon materials can be finely tuned and thus generate abundant catalytic active sites for ORR/OER.

**Figure 11:**
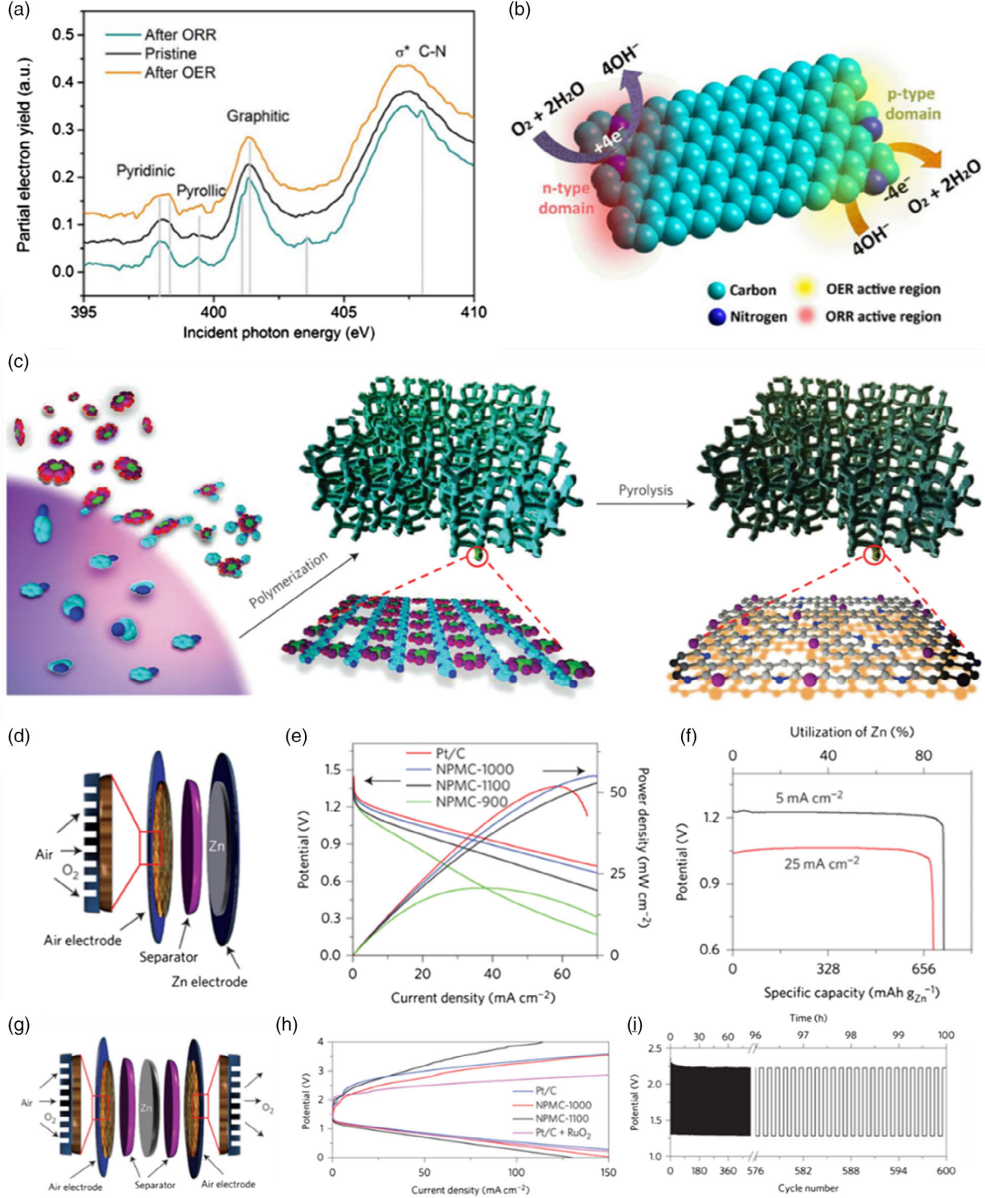
a) The N K‐edge XANES spectra of N‐GRW. b) ORR and OER occurring at different active sites of the NGRW catalyst. Reproduced with permission.^[^ ^76^ ^]^ Copyright 2016, American Association for the Advancement of Science. c) The NPMC foams preparation process. d–f) Performance of primary ZABs using the NPMC foams. g–i) Performance of rechargeable ZABs using the NPMC foams. Reproduced with permission.^[^ ^77^ ^]^ Copyright 2015, Springer Nature.

N‐doping seems to be the most efficient method compared with other heteroatoms doping because of its higher electronegativity than C. When the carbon materials are doped with N, the positive charge will be created around carbon atoms, which can improve the oxygen chemisorption and electron transfer, thus resulting in the enhanced catalytic activities for ORR/OER.^[^ ^74^ ^]^ N can exist in the form of graphitic, pyridinic, pyrrolic, and oxide forms when incorporated into the graphitic network.^[^ ^75^ ^]^ Recently, Dai's group reported a 3D structured N‐doped graphene nanoribbon (N‐GRW),^[^ ^76^ ^]^ which contains both p‐type and n‐type N dopant configurations. The N‐GRW was used as catalysts for ORR and OER. From the X‐ray absorption near edge structure (XANES) spectra of N K‐edge, they found that the quaternary N peak after ORR was formed, while the intensity of pyridinic N peak increased after OER (**Figure** **11**a). The n‐type doping quaternary N was identified as the active sites for ORR, while the p‐type doping pyridinic N in the N‐GRW acted as active sites for OER (Figure 11b). The carbon materials doped with other heteroatoms also show good ORR/OER performance, such as P, S, and Se (a bigger atomic radius and more electronegative than C) and B, F (a smaller atomic radius and less electronegative than C). In addition to single heteroatom‐doped carbon, the binary heteroatom‐doped (such as N/P, N/S, N/F, N/P) and ternary heteroatom‐doped (such as N/P/B and N/F/B) carbon materials also have been investigated to improve the ORR/OER performance. In future research, more advanced techniques should be developed to precisely identify the active sites of these heteroatom‐doped carbon materials.

Dai's group also reported N and P codoped mesoporous carbon foam (NPMC).^[^ ^77^ ^]^ The NPMC with a super high surface area (1663 m^2^ g^−1^) was prepared by pyrolysis of polyaniline aerogel containing phytic acid (Figure 11c). The pyrolysis temperature is very important because the relatively low pyrolysis temperature can result in high charge‐transfer resistance. However, the overheating at high pyrolysis temperature will lead to the removal of doped heteroatoms (N and P), resulting in the reduced active sites. They found that the optimal temperature is 1000 °C, at which the moderate graphitization degree, electrical conductivity, surface area, and N/P content can be well kept. The metal‐free NPMC can be used as air cathodes for both primary ZABs (Figure 11d–f) and rechargeable ZABs (Figure 11g–i). Specifically, the OCP of primary ZABs can be as high as 1.48 V with a peak power density of 55 mW cm^−2^. The specific capacity and energy density of 735 mAh g_Zn_^−1^ and 835 Wh kg_Zn_^−1^ (Figure 11d–f), respectively, were achieved and stably operated at 2 mA cm^−2^ for 240 h with just replacing new Zn anode (mechanical recharging). It shows stable performance for 600 cycles (100 h) in rechargeable ZABs at 2 mA cm^−2^ in three‐electrode ZABs (Figure 11g–i). According to the DFT calculations, it is found that the synergistic electronic interactions are generated by N, P dopants, and adjacent carbon atoms help improve the electrocatalytic bifunctional OER and ORR activities.

Based on the aforementioned discussions, the modified carbon‐based materials are widely used as support/active materials for air cathodes in ZABs due to their high conductivity, porosity, and high specific surface area. However, these carbon materials suffer from serious and rapid structure decays and are easily oxidized to carbon monoxide/carbon dioxide under anodic oxidizing conditions under the high applied voltage during the charging process. This is bound to result in the poor stability and inferior circulation of air electrodes during the charge/discharge process in rechargeable ZABs, and in turn, reduce the catalytic activities due to the collapse of the carbon structure and the dissolution/aggregation/demetalation of the metals on the carbon supports. Although the increased graphitization degree of carbon materials can inhibit the corrosion to a certain extent, the lifetimes of carbon materials can still be significantly reduced due to the electrochemical corrosion with the strong alkaline solution. Thus, exploring materials with anticorrosion, high electrical conductivity, and high surface area is necessary. The metal foam/mesh (such as Ni and Cu) seems to be effective alternative supports (Figure 6c). In all, much effort should be paid attention to these carbon corrosion concerns.

#### 4.2.4 Carbon‐Free Catalysts

As stated earlier, high operation potential is needed during the charging process in ZABs. However, the standard thermodynamic potential for carbon oxidation to CO_2_ under alkaline solution is 0.621 V (vs SHE, Equation (11)),^[^ ^78^ ^]^ which is much lower than the charging voltage of ZABs (usually exceed 2.0 V). Thus, the carbon‐based materials are easily oxidized, and most of the OER catalytic materials are metal or metal oxides (Figure 7c).

(11) $$C+4{\text{OH}}^{-}\to {\text{CO}}_{2}+2H_{2}O+4e^{-}\;(E^{0}=0.621\text{ V vs SHE})$$

**Figure 12:**
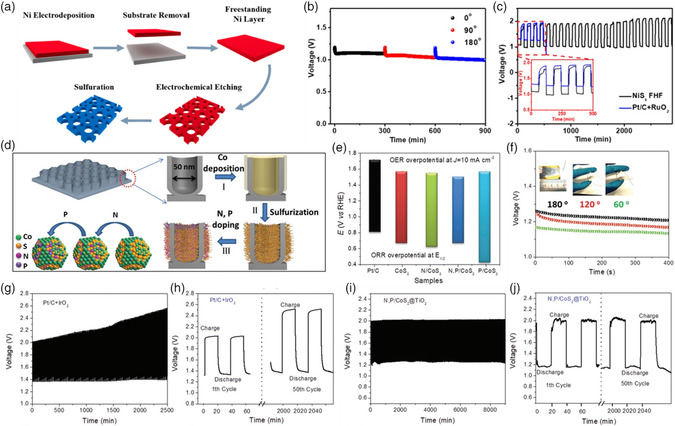
a) Fabrication process of freestanding NiS_*x*_ FHF. b) Galvanostatic discharge curves of the flexible ZAB with different deformation angles (0–180°) using NiS_2_ FHF as the air cathode at 2 mA cm^−2^; c) galvanostatic charge–discharge performance for flexible ZABs using NiS_*x*_ FHF and Pt/C + RuO_2_ as air electrode, respectively. Reproduced with permission.^[13h]^ Copyright 2018, American Chemical Society. d) The fabrication process of the N,P/CoS_2_@TiO_2_ NPFs; e) overall bifunctional ORR/OER performances of N,P/CoS_2_@TiO_2_ NPFs; f) galvanostatic discharge performance of ﬂexible ZABs with different deformation angles (60–180°). g–j) Discharge–charge profiles of ZABs with and g–h) Pt/C + IrO_2_ and i–j) N, P/CoS_2_@TiO_2_ catalysts. Reproduced with permission.^[^ ^79^ ^]^ Copyright 2018, Wiley.

The porous metal (alloy) films, spinel, rutile, perovskite have been widely studied due to the low cost, high abundance, and environmental friendliness. To solve the shortcomings of carbon‐based materials that being easily oxidized and corroded in electrochemical ORR/OER environments, a novel strategy combining electrodeposition with electroetching was reported to prepare an additive‐free nickel sulfide (NiS_*x*_) freestanding holey film (NiS_*x*_ FHF, **Figure** **12**a).^[13h]^ The NiS_*x*_ with porous structure has an optimal electrochemically active surface area and active sites, which is a good candidate as stable bifunctional ORR/OER catalysts and can be directly used as air cathode. The stability and potential application of the flexible rechargeable ZABs using NiS_*x*_ FHF were assessed by the galvanostatic discharge curves with different deformation angles at a current density of 2 mA cm^−2^. Even with different deformation angles, the flexible ZABs show no obvious performance decay (Figure 12b), thus validating the potential application of NiS_*x*_ FHF as air cathodes for flexible ZABs. In addition, galvanostatic charge–discharge curves further confirm the much better stable performance of NiS_*x*_ FHF than benchmark Pt/C‐RuO_2_ electrodes (Figure 12c). This work provides an effective strategy to prepare carbon‐free, PGMs‐free, and nonadditive freestanding porous electrodes for ZABs.

As discussed earlier, apart from boosting the performance of carbon‐based materials, heteroatoms doping strategy can also be applied to the metal‐based materials to enhance the conductivity, increase the active surface area, and facilitate reaction kinetics. For example, the N, P‐codoped CoS_2_ nanoclusters embedded inside TiO_2_ nanoporous films (N, P/CoS_2_@TiO_2_ NPFs) were developed,^[^ ^79^ ^]^ as shown in Figure 12d. After the anodic oxidization treatment, TiO_2_ NPFs with a highly ordered honeycomb morphology were obtained, which were subsequently coated by Co and processed sulfurization treatment to construct a high active bifunctional ORR/OER catalysts. Compared with the benchmark Pt/C and other control samples, the N, P/CoS_2_@TiO_2_ NPF shows the lowest voltage gap between *E*_1/2_ for ORR and *E*_onset_ (10 mA cm^−2^) for OER (0.78 V, Figure 12e). The as‐prepared N, P/CoS_2_@TiO_2_ NPFs can be directly used as air cathodes for flexible ZABs. The galvanostatic discharge curves of ﬂexible ZABs using N, P/CoS_2_@TiO_2_ NPFs at different deformation angles show no obvious performance decay at 10 mA cm^−2^, indicating the great potential application in flexible devices (Figure 12f). Moreover, compared with the gradual performance decay (the voltage gap was increased over time) of ZABs using Pt/C‐RuO_2_ as air cathodes (Figure 12g,h), the N, P/CoS_2_@TiO_2_ NPFs‐based ZABs also showed strong stability even with a continuous 200 cycles (133 h) test (Figure 12i,j). Furthermore, the specific capacity of 610 and 580 mAh g Zn^−1^ was obtained at a discharge current density of 10 and 50 mA cm^−2^. From the in‐depth analysis, it is found that the heteroatoms play different roles in the N, P/CoS_2_@TiO_2_ NPFs, that is, N‐doping was mainly used to increase the conductivity and electrochemical activity of the NPFs, and the P‐doping can provide the electrode a passivated surface and thus improve stability. Thus, this N, P/CoS_2_@TiO_2_ NPFs show outstanding ORR/OER performance for ZABs.

**Figure 13:**
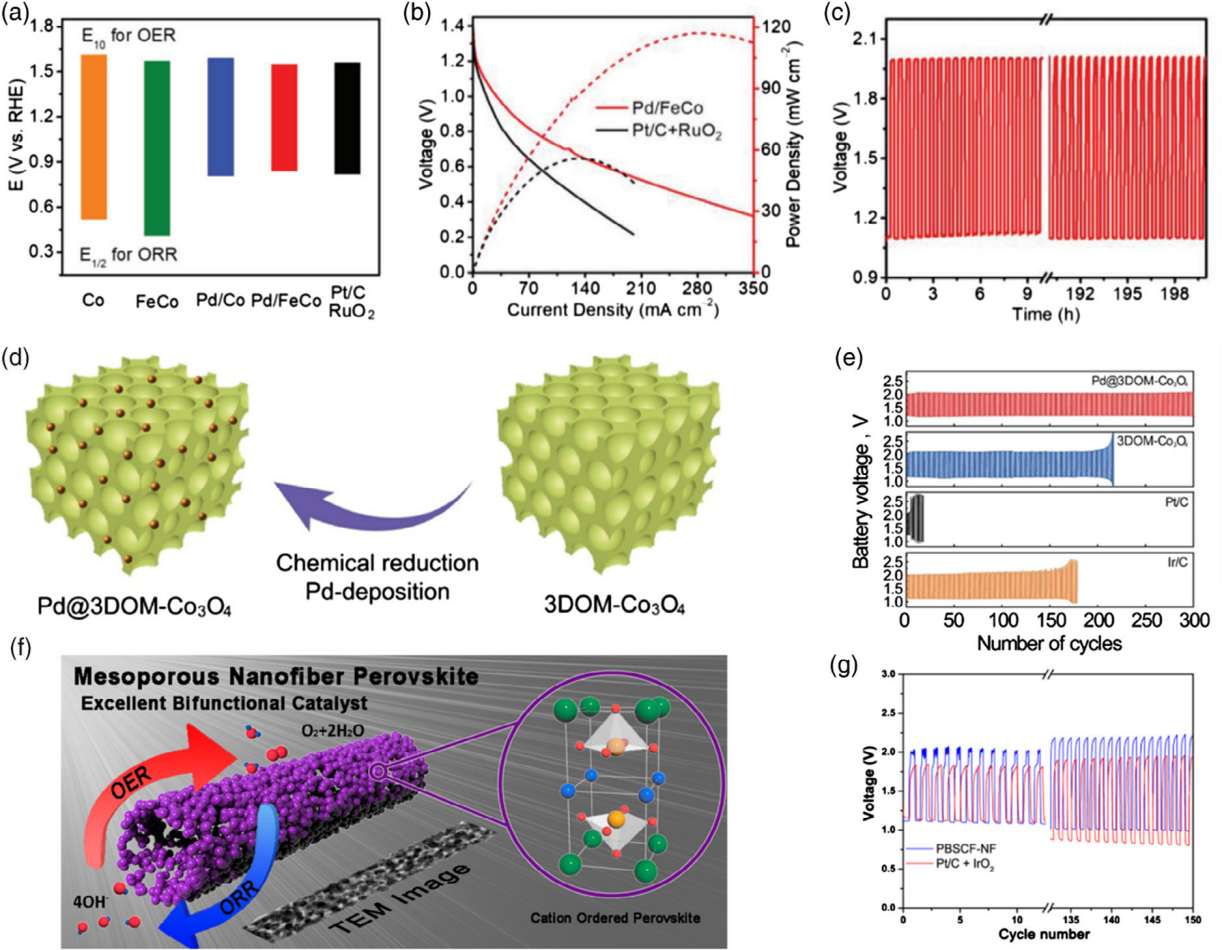
a) Comparison of bifunctional ORR/OER performance of Pd/FeCo and control samples. b,c) Rechargeable ZABs performance of Pd/FeCo. Reproduced with permission.^[58n]^ Copyright 2020, Wiley. d) Synthesis of Pd@3DOM‐Co_3_O_4_, e) rechargeable ZABs performance using Pd@3DOM‐CO_3_O_4_. Reproduced with permission.^[^ ^85^ ^]^ Copyright 2018, Elsevier. f) Schematic of the cation‐ordered PBSCF‐NF perovskite as a bifunctional ORR/OER catalyst. g) The rechargeable ZABs performance of PBSCF‐NF at a current density of 10 mA cm^−2^. Reproduced with permission.^[^ ^101^ ^]^ Copyright 2017, American Chemical Society.

The free‐standing metal porous films can be used not only as catalysts, but also as support to replace carbon materials due to their good conductivity and corrosion resistance. Recently, a porous FeCo glassy alloy film was developed as bifunctional catalytic support to replace conventional carbon supports. The conducting framework of the porous FeCo glassy alloy was used to stabilize ORR/OER cocatalysts, in which the FeCo was the main active site for OER, and the ultrasmall Pd nanoparticles anchored on the FeCo glassy alloy as ORR active sites. A half‐wave potential of 0.85 V (vs RHE) for ORR was acquired for the Pd/FeCo, which is almost the same as Pt/C. Also, it only needs 1.55 V (vs RHE) to reach the current density of 10 mA cm^−2^ for OER (**Figure** **13**a). The Pd/FeCo can be directly used as the air cathode in rechargeable ZABs, exhibiting a maximum power density of 117 mW cm^−2^ (Figure 13b) and no obvious performance attenuation after continuous operation for 400 cycles (200 h, Figure 13c). This work offers a new strategy to design novel carbon‐free self‐supported materials for ZABs.

Due to the possessed multiple valence states, some transition metal (i.e., Fe, Co, Ni, and Mn) oxides have drawn much attention. For example, the variable valences of Mn and abundant structures of MnO_*x*_ endow the Mn‐based materials with favorable redox electrochemistry, thus providing a wide opportunity to seek and develop non‐PGMs and carbon‐free materials. All the MnO_2_, Mn_2_O_3_, Mn_3_O_4_, Mn_5_O_8_, and even MnOOH exhibit ORR activities in alkaline solution.^[^ ^80^ ^]^ For example, Jaramillo reported a simple and effective electrodeposition approach together with a thermal treatment in the air to prepare nanostructured MnO_*x*_ thin‐film materials on glassy carbon.^[^ ^81^ ^]^ The MnO_*x*_ thin film shows comparable ORR and OER performance to the commercial Pt/C, Ir/C, and Ru/C. For the MnO_2_, it has three crystal structures, and the catalytic ORR activities increase in the order of γ‐MnO_2_ < β‐MnO_2_ < α‐MnO_2_.^[^ ^82^ ^]^ One trend of the Mn‐based materials for ORR is that the catalytic activities correlate with the nanostructures, exposed facets, and Mn valence. However, much more efforts should be taken to explore the in‐depth structure–valence–performance relationship of Mn‐based materials.

Co_3_O_4_ has been widely used as OER catalysts;^[^ ^83^ ^]^ however, its ORR performance is poor and rarely studied. Coupling Co_3_O_4_ with other materials, such as Ni, Mn, and Pd, offers an effective method to improve ORR performance through electronic effect.^[^ ^84^ ^]^ For example, Chen's group reported chemical deposition of Pd nanoparticles on the 3D ordered mesoporous Co_3_O_4_ (Pd@3DOM‐CO_3_O_4_, Figure 13d).^[^ ^85^ ^]^ The d‐band center of Pd was decreased, while the Fermi level was increased due to the role of 3DOM Co_3_O_4_, which improved the overall kinetics and conductivity in ZABs. The Pd@3DOM‐CO_3_O_4_ was used as an air cathode in rechargeable ZAB, showing long‐term stability of 300 cycles (50 h, Figure 13e).

Other bifunctional oxides were also studied, such as spinel (A_*x*_B_3—*x*_O_4_) structure and perovskite (ABO_3_) structure.^[^ ^86^ ^]^ All these materials can be used as air cathodes due to their high catalytic activity for ORR/OER and excellent anticorrosion property in alkaline solution. Recent studies indicate that the ORR activities of perovskite materials are related to the s*‐orbital (e_g_) occupation and transition metal–oxygen covalency, which can be used as activity descriptors (Figure 7c).^[^ ^87^ ^]^ Kim et al. reported a mesoporous nanofibers cation‐ordered perovskite, PrBa_0.5_Sr_0.5_Co_2—*x*_Fe_*x*_O_5+*δ*_ (*x* = 0, 0.5, 1, 1.5, and 2), in which the B‐site metal ratios and the surface of mesoporous nanofibers can be well controlled (Figure 13f). This cation‐ordered perovskite showed high bifunctional activities for ORR/OER and good stability in ZABs (Figure 13g). Chen et al. reported a bifunctional ORR/OER catalyst with a core‐corona structure (CCBC) consisting of LaNiO_3_ and N‐doped carbon nanotubes.^[^ ^88^ ^]^ In these CCBC materials, the core was responsible for OER, while the corona was designed to catalyze ORR. When tested in ZABs at 17.6 mA cm^−2^, a voltage gap of 1.3 V was achieved. Other types of perovskites, such as La_1.7_Sr_0.3_NiO_4_, LaMO_3_ (M = Ni, Co, Mn), and Ba_0.6_Sr_0.4_Co_0.79_Fe_0.21_O_2.67_, were also reported for ZABs.^[^ ^89^ ^]^

## 5 Summary and Perspectives

**Figure 14:**
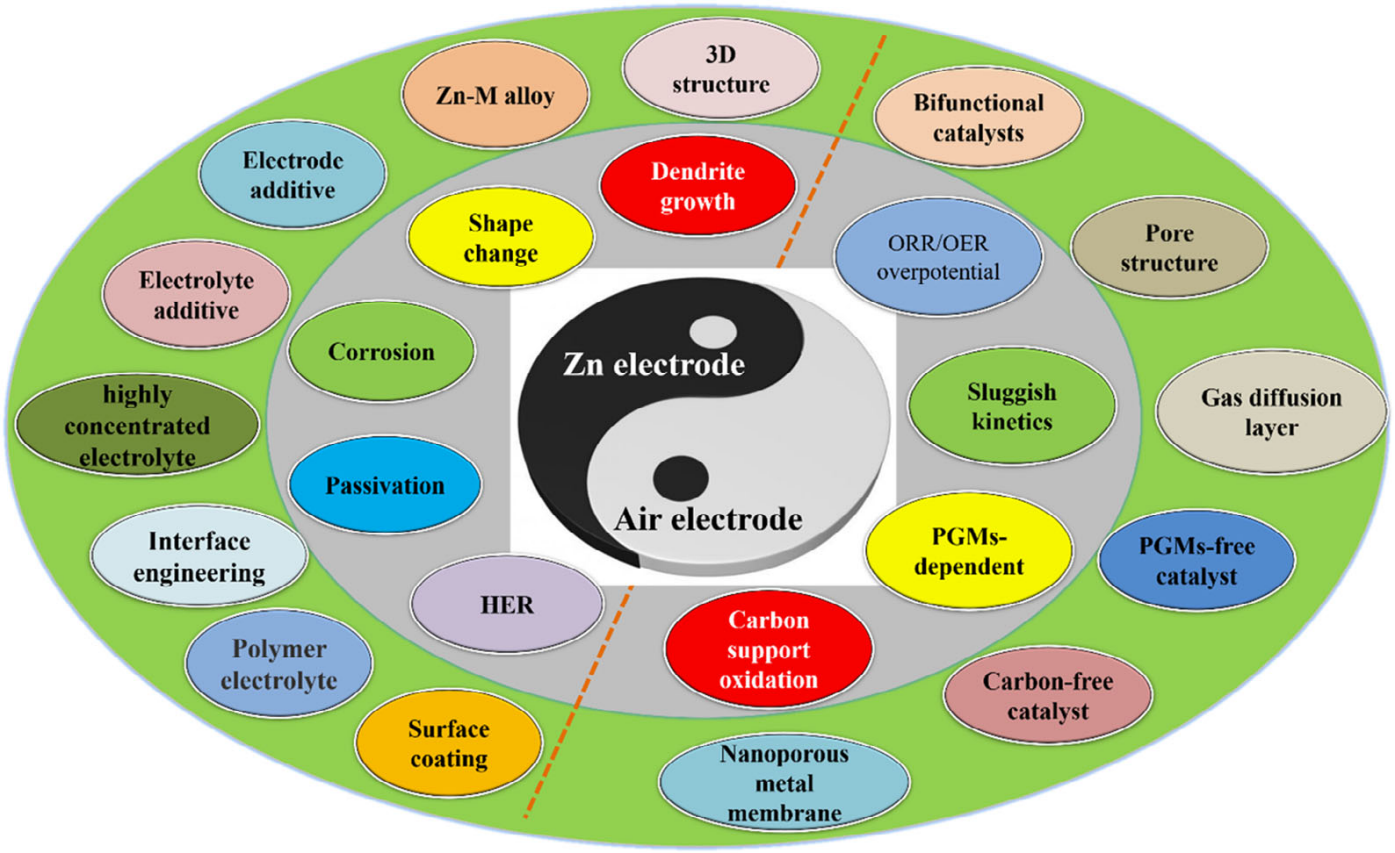
The problems on the Zn anode and air electrode, and the effective solutions for ZABs.

In this review, we systematically summarize the recent progress in electrode design from perspectives of electrochemistry and material science. The problems on the Zn anode, such as dendritic growth, shape reconstruction, passivation, and HER, were critically reviewed, and some effective solutions were proposed. For the air cathodes, the advantages and limitations of different types of bifunctional ORR/OER electrocatalysts, including PGMs, carbon‐based materials, and carbon‐free materials, have been summarized in **Figure** **14** and Table 1. The electrocatalytic reaction mechanism, influential factors, and electrode performance of ZABs were emphasized in this review. Although great efforts and some outstanding research achievements have been accomplished, the development of ZABs remains confronted with severe challenges.

1) New working mechanisms for ZABs. Many fundamentals of electrochemistry and material science still need thorough understanding. For example, the 4e^−^ transfer process of Zn anode was recognized as the best choice with high efficiency at the beginning of the ZABs study. However, the ZnO_2_/Zn with a 2e^−^ transfer process was proposed recently,^[^ ^90^ ^]^ which shows better reversibility during continuous charge and discharge process. In future studies, more strategies should be developed to improve the rate performance and power density of ZnO_2_/Zn batteries. Also, it is highly recommended to have a further understanding of the underlying mechanism of Zn redox chemistry. In addition, the dendrite growth, self‐discharge, HER on the Zn electrode should also be addressed. Although some effective strategies such as using flow cells to mitigate the anode issues, the flowing system cannot be used in flexible devices. In addition, extra tubes, pumps, and excessive electrolytes are needed for flow cells. Extra electrical energy is also needed to drive the pump, which will not only increase the system complexity, volume, and cost but also decrease the energy efficiency. From material science aspects, increasing Zn anode surface area and fabricating 3D Zn structure have been used to improve the ZABs cyclability and Zn utilization efficiency. However, the increased Zn surface area will lead to severe HER on the electrode, which further reduces the performance of ZABs. Thus, the development of novel Zn structure, architecture, and alloy strategies is highly desired, and new mechanisms for ZABs could be further investigated.

2) New electrolytes. Although the alkaline aqueous electrolyte‐based batteries show the best activity under ZABs operation conditions, the possible leakage and evaporation of alkaline electrolytes will corrode human skin and instruments when used for flexible electronics. In addition, the ZABs performance is largely dependent on the pH of the electrolytes. As the most commonly used electrolyte is a strong alkaline, it is recommended to real‐time monitor the pH changes in the ZABs operation, especially during the charge and discharge process. Current experimental methods only report the pH of electrolytes before and after the operation, while important and useful information during the operation is not easily accessed. The pH value of electrolytes can determine the surface change, structure transformation, and active site formation/evolution/depopulation of Zn anodes and air cathodes. Thus, real‐time monitoring of the electrolyte pH is important for designing electrode materials. In addition, a solid‐state electrolyte is preferred in flexible ZABs. However, the much lower ionic conductivity of solid‐state electrolytes than aqueous electrolytes results in the inferior performance of flexible ZABs, and it is difficult to real‐time monitor the pH changes for the solid‐state electrolyte. In summary, the development of solid‐state electrolytes and ionic liquids with low price and high ionic conductivity must be considered when extending the practical application of flexible ZABs.

3) Water consumption in aqueous electrolyte. Most of the alkaline aqueous electrolytes are prepared by deionized water and freshwater on a laboratory scale. However, the freshwater resources shortage currently urges us to search for alternative water resources, such as seawater, low‐grade and saline surface water, which is almost free and inexhaustible on earth.^11^, ^91^ However, for seawater‐based ZABs, there are large numbers of anions (mainly Cl^−^) and cations (mainly Na^+^, K^+^, Ca^2+^, Mg^2+^) in seawater. In addition, the biofouling and trace metal deposition also happen in seawater and saline water. Though the insoluble impurities can be removed by the filter, the Na^+^, K^+^, and Cl^−^ cannot be removed and are still existed in seawater. The Cl^−^ ions will result in chlorine evolution reaction (ClER),^[^ ^92^ ^]^ which is a competitive reaction of OER during the charging process due to the very close thermodynamic potentials for OER and ClER.^[^ ^93^ ^]^ Thus, the seawater purification and development of catalysts with high selectivity for OER will be recommended.^[^ ^94^ ^]^ The effects of both the anions and cations on the seawater‐based ZABs performance should be carefully studied.

4) CO_2_ and H_2_O managements. During the discharging process, the cathode materials need to react with O_2_ from the atmosphere for ORR. During the air diffusion into the air cathode, CO_2_ and H_2_O vapor will also diffuse into the electrolyte. Consequently, CO_2_ will react with alkaline electrolytes and thus generate carbonate to block the active sites of electrocatalysts and gas diffusion channels. And H_2_O vapor will change the pH value of the electrolyte. Both CO_2_ and H_2_O vapor should be filtered out before the ambient air diffusing into the electrolyte. Accordingly, adding a membrane to the air cathode to permit O_2_ diffusion selectively instead of the incorporation of CO_2_ and H_2_O is a feasible approach to control CO_2_ and H_2_O diffusion (Figure 6c). Some researchers also recommended adding a ‘‘scrubber’’ using cheap hydroxides (such as Ca(OH)_2_, soda lime, and amines).^[^ ^95^ ^]^ Nevertheless, all the strategies will increase the system cost and complexity. Developing CO_2_‐ and H_2_O‐resistant electrolytes and using acidic/neutral electrolytes should be considered.^[^ ^96^ ^]^

5) Surface wettability of GDL and CL. To avoid water flooding and keep the well‐controlled O_2_ diffusion, a hydrophobic GDL should be used. While at the same time, to enlarge the three‐phase reaction interface and guarantee the intimate contact of gas–electrolyte–catalyst, a hydrophilic CL is also needed. The well‐balanced hydrophobic and hydrophilic properties of GDL and CL should be seriously considered. The PTFE‐treated carbon paper (or carbon cloth) is a good choice for GDL, while the serious electrochemical oxidation corrosion of carbonaceous materials under high applied potential (>2.0 V) will result in the gradually poisoned and deactivated carbon paper, leading to performance decay of ZABs. The porous metallic films and foams, foils, and sponges can be used as succedaneums for carbon‐based materials, which have good conductivity and stability under corrosion potential. However, the surface/interface of these metal films, foams, foils, and sponges should be further used by hydrophobic treatment to inhibit electrolyte flooding. Thus, the rational design of the air cathodes with optimal hydrophobic/hydrophilic properties and friendly interface structure/architecture is important.

6) Development of high‐performance, cost‐effective, anticorrosion, and stable bifunctional catalysts. PGMs‐based and carbon‐based catalysts have been widely used in the development of air cathodes for ZABs. The high cost of PGMs limits their commercial applications in ZABs, while the carbon corrosion leads to poor cycling stability of carbon‐based ZABs. Although great efforts have been made for the development of ORR and OER electrocatalysts, the inherent incompatibility of ORR and ORR due to the separated vertices of the ORR/OER volcano plots (Figure 7d) makes it difficult to develop bifunctional ORR/OER catalysts with high performance and stability. Most of the reported rechargeable ZABs can only be stably operated from tens of hours to hundreds of hours, and only a few works can be operated for more than 1000 h (Table 1). The flexible ZABs using solid‐state electrolyte show much faster performance decay due to the gradual consumption of electrolyte even operated under a small current density. These performance of reported ZABs are far from the practical application (typically at least thousands of hours or even tens of thousands of hours). Thus, developing air electrodes with high performance, cost‐effectiveness, and anticorrosion is highly desired. The porous metals and alloys are good options for ZABs, which can be used as GDL and CL simultaneously. However, the balanced hydrophobic/hydrophilic properties, cost, usage amount, and surface area should be studied.

Overall, the excellent superiorities of safety, high energy density, and low cost allow the ZABs as a green and low‐carbon energy storage and conversion technology. Much more scientific and industrial efforts on the developments of ZABs are highly encouraged. The ZABs will be ultimately used in many fields including wearable and portable devices.

## Conflict of Interest

The authors declare no conflict of interest.
